# ATF4/CEMIP/PKCα promotes anoikis resistance by enhancing protective autophagy in prostate cancer cells

**DOI:** 10.1038/s41419-021-04494-x

**Published:** 2022-01-10

**Authors:** Ying Yu, Bing Liu, Xuexiang Li, Dingheng Lu, Likun Yang, Liang Chen, Yunxue Li, Lulin Cheng, Fang Lv, Pu Zhang, Yarong Song, Yifei Xing

**Affiliations:** 1 Department of Urology, Union Hospital, Tongji Medical College, Huazhong University of Science and Technology, Wuhan, 430022 China; 2 Department of Critical Care Medicine, Union Hospital, Tongji Medical College, Huazhong University of Science and Technology, Wuhan, 430022 China; 3 Present Address: Department of Urology, Zhongnan Hospital of Wuhan University, Wuhan, 430061 China

**Keywords:** Metastasis, Autophagy

## Abstract

The survival of cancer cells after detaching from the extracellular matrix (ECM) is essential for the metastatic cascade. The programmed cell death after detachment is known as anoikis, acting as a metastasis barrier. However, the most aggressive cancer cells escape anoikis and other cell death patterns to initiate the metastatic cascade. This study revealed the role of cell migration-inducing protein (CEMIP) in autophagy modulation and anoikis resistance during ECM detachment. CEMIP amplification during ECM detachment resulted in protective autophagy induction via a mechanism dependent on the dissociation of the B-cell lymphoma-2 (Bcl-2)/Beclin1 complex. Additional investigation revealed that acting transcription factor 4 (ATF4) triggered CEMIP transcription and enhanced protein kinase C alpha (PKCα) membrane translocation, which regulated the serine70 phosphorylation of Bcl-2, while the subsequent dissociation of the Bcl-2/Beclin1 complex led to autophagy. Therefore, CEMIP antagonization attenuated metastasis formation in vivo. In conclusion, inhibiting CEMIP-mediated protective autophagy may provide a therapeutic strategy for metastatic prostate cancer (PCa). This study delineates a novel role of CEMIP in anoikis resistance and provides new insight into seeking therapeutic strategies for metastatic PCa.

## Introduction

Escaping anoikis, a form of programmed cell death, is critical for cancer cell survival after detaching from the extracellular matrix (ECM). Anoikis resistance ensures the survival of aggressive cells in the circulatory system and is deemed essential for cancer progression [1]. Although various studies have revealed that cancer cells utilize multi-faceted mechanisms to evade anoikis [1, 2], the precise molecular mechanisms involved in prostate cell survival following ECM detachment remains unclear.

ECM detachment is a physiological trigger for autophagy that can cause cell death (autophagic cell death) or maintain cell survival during nutrient deficiency by recycling intracellular components to generate energy [3–7]. The exact role of autophagy in prostate cancer (PCa) cell survival in nonadherent conditions and whether autophagy is linked with anoikis resistance remain unclear. Previous studies have suggested that the disruption of anoikis resistance and autophagy might serve as a therapeutic strategy for advanced PCa [8, 9]. However, few molecular targets link protective autophagy with anoikis resistance in PCa cells. To identify the potential oncogenes that regulate anoikis in PCa cells, this study establishes an anoikis-resistant PCa cell model (PCa-AR) via the continuous culturing of human PCa cell lines in suspension conditions [10, 11]. The results reveal that cell migration-inducing protein (CEMIP), also known as KIAA1199, is significantly overexpressed in PCa-AR cells, promoting tumor metastasis via metabolic reprogramming [12].

Elevated CEMIP is evident in malignancies, while CEMIP suppression impairs tumor growth, epithelial-mesenchymal transition, and metastasis [13–15]. The CEMIP in the endoplasmic reticulum (ER) mediates calcium leakage, resulting in cytosolic calcium accumulation. Increased cytosolic calcium may lead to the translocation and activation of PKCα, activating the downstream signaling pathways responsible for cancer cell migration [16]. When cells undergo anoikis, activated ER stress can induce autophagy for cell survival via several different mechanisms [9, 17–20].

This study shows that CEMIP enhances autophagy and protects PCa cells from anoikis in nonadherent conditions. Mechanistically, it is demonstrated that ATF4 directly binds to CEMIP 3′UTR to promote CEMIP transcription. More specifically, CEMIP mediates calcium leakage from the ER, causing PKCα translocation. Cell membrane PKCα enhances autophagy by promoting B-cell lymphoma-2 (Bcl-2)/Beclin1 complex dissociation, leading to PCa cell anoikis resistance. The results highlight the novel role of CEMIP in ECM-detached cell survival and provide a novel therapeutic strategy for advanced PCa treatment.

## Materials and methods

### Patient samples for clinicopathological characteristics analyses

A total of 60 sets of specimens of PCa tissues and their adjacent normal prostate tissues were obtained from patients who underwent radical cystectomy for prostate carcinoma at the Department of Urology of Union Hospital affiliated of Tongji Medical College between 2015 and 2019. We had acquired the approval from the Institutional Review Board of Tongji Medical College of Huazhong University of Science and Technology before we collected the samples. All patients have signed an informed consent form. Consent was obtained for publication of patient photos. All specimens were classified according to the 2004 World Health Organization Consensus Classification and Staging System for prostate neoplasms.

### Cell culture and cell lines

Cells were maintained in RPMI 1640 medium (Hyclone, GE Healthcare Life Sciences, Logan, UT, USA) with 10% fetal bovine serum (Biologic Industries, Kibbutz Beit Haemek, Israel) and 1% penicillin/streptomycin (Beyotime Institute of Biotechnology, Nanjing, China, C0222) at 37 °C in 5% CO_2_ and 95% humidified air. To establish the anoikis-resistant model, corresponding parental cells were continuously cultured in ultra-low-attachment six-well plates (Corning, NY, USA) for 7 days and then transferred to normal plates to allow adherence for 24 h. Re-adherent cells were deemed anoikis-resistant [9, 10, 16]. Cell culture and the anoikis-resistant model were established by using human androgen-independent PCa cell lines, PC-3 and DU145, which were obtained from Shanghai Cell Bank, Chinese Academy of Sciences (Shanghai, China). Androgen-independent C4-2 and androgen-dependent LNCaP cell lines were gifts from Prof. Xiaoping Zhang and Prof. Jun Zhao (Union Hospital, Wuhan, China).

### Mouse models

All animal experiments were approved by the Animal Care Committee of Tongji Medical College. We chose 4-week-old male BALB/c nude mice for tumor xenografts experiments. Additional information on experimental methods in next section.

### Detection of apoptosis

Apoptosis of non-transfected cells was detected using the FITC-Annexin V apoptosis detection kit (BD Biosciences, Franklin Lakes, NJ, USA, 556547), and the PE-Annexin V apoptosis detection kit (BD Biosciences, Franklin Lakes, NJ, USA, 559763) was used to detect transfected cells. In brief, cells (5 × 10^5^) were collected and incubated with FITC/propidium iodide or PE/7-Amino-Actinomycin D (BD Biosciences, Franklin Lakes, NJ, USA, 559925) for 15 min in the dark at room temperature, and the apoptosis index was determined via flow cytometer (Beckman Coulter, Indianapolis, IN, USA). For the detachment-induced apoptosis assay, cells were incubated in ultra-low-attachment plates for 48 h before detection.

### Cell migration and invasion assays

Cell migration was evaluated by using 24-well transwell plates with 8.0-mm pore polycarbonate membrane inserts (Corning, NY, USA). For cell invasion, chamber inserts were coated with 50 μl Matrigel (BD Biosciences). Homogeneous single-cell suspensions (200 μl; 1 × 10^5^cells/well) in serum-free medium was added to the upper chambers and 500 μl complete medium was added to the lower chambers. After incubation for 24 h at 37 °C in a CO_2_ incubator, migratory or invasive cells were fixed with ice-cold methanol and stained with 0.1% crystal violet for 15 min at room temperature. Migratory or invasive cells were counted in 3 randomly chosen fields under an inverted phase-contrast microscope (Olympus, Tokyo, Japan) at ×200 magnification.

### Quantitative real-time PCR

cDNA was synthesized from total RNA using the iScript cDNA synthesis kit (Bio-Rad, Hercules, CA, USA) following manufacturer protocols. Quantitative real-time PCR was performed by the ABI Power SYBR Green PCR Master Mix (Applied Biosystems, Foster City, CA, USA) and the 7900 HT Sequence Detection System (Applied Biosystems). Glyceraldehyde 3-phosphate dehydrogenase was used as internal control. PCR primer pairs were synthesized by Sangon Biotech (Shanghai, China), and primer sequences are presented in Supplementary material Table 1.

### Membrane and cytosol protein extraction kit

Membrane and cytosol protein of PKCα in this study were isolated by using Membrane and Cytosol Protein Extraction Kit (Beyotime Institute of Biotechnology, P0033). The distribution ratio and change of PKCα in cell membrane and cytoplasm were detected by western blot assay.

### Western blot

Total cellular protein was isolated by using a RIPA lysis buffer (Beyotime Institute of Biotechnology, Shanghai, China, P0013E), separated on SDS-PAGE gels, and transferred to PVDF membranes (EMD Millipore, Billerica, MA, USA). Membranes were blocked by 5% nonfat milk in Tris-buffered saline with Tween-20, then incubated with primary Abs overnight at 4 °C and with corresponding secondary Abs afterwards (ProteinTech, Chicago, IL, USA). Detailed information about the antibodies used in this study can be found in the Supplementary material Table 2. Protein bands were visualized with ECL (Beyotime Institute of Biotechnology, P0018S).

### Transmission electron microscope

Cells were collected and fixed with 2.5% glutaraldehyde at 4 °C for 2 h. The ultrastructure of autophagy and autolysosome in cells were observed using a transmission electron microscope (TEM).

### Tissue immunohistochemistry

Tissue samples from 60 patients with PCa were obtained from the Department of Urology, Wuhan Union Hospital. Immunohistochemistry (IHC) was conducted as described previously [21]. IHC staining was performed by using CEMIP Ab (1:500; Abcam). Immunoreactivity was scored based on a combination of both the percentage and intensity of positively stained tumor cells to generate an H-score. To compare the expression of CEMIP with clinicopathological characteristics, samples were grouped in high and low-expression groups. In addition, in this study, the distribution and expression levels of ATF4, CEMIP, PKCα, and Beclin1 were detected by immunohistochemistry in the lung metastasis tissues of nude mice in animal experiments. The information of the antibodies used is detailed in Supplementary material Table 2. Cut off value was determined by median of H-score. IHC images were photographed at ×400 magnification.

### Treatment of autophagy activator and inhibitors

Anoikis-resistant PC-3 and DU145 cells were treated with 200 nM rapamycin (rapa) (Selleck Chemicals, Houston, TX, USA, S1039), 10 mM 3-Methyladenine (3-MA) (Selleck Chemicals, S2767) for 48 h, respectively [22–25]. Isometric double-distilled water or DMSO was used as negative control. Details of small molecule reagents and kits used in this study are shown in Supplementary materials Table 3.

### Cell viability assay

Control group cells and treatment group cells were plated on 96-well plates at 5000 cells/well and cultured with complete medium overnight. After 24 h, 10 ml of Cell Counting Kit-8 solution (Dojindo Laboratories, Kumamoto, Japan) was added to each well and the plate was incubated for 2 h at 37 °C. Absorbance at 450 nm was measured on a microplate reader (Tecan, Mannedorf, Switzerland). This process was repeated for seven days.

### RNA interference

For Bcl-2 silencing, anoikis-resistant PCa cells were seeded on six-well plates at 50% confluency with an antibiotic-free complete medium. The next day, 1 μg of the Bcl-2 siRNA duplex (Santa Cruz Biotechnology, Dallas, TX, USA) or control siRNA plus 8 μl of transfection reagent (Santa Cruz Biotechnology) was diluted in 100 μl of transfection medium. The resultant mixture was incubated for 30 min at room temperature. Transfection mixtures were added to the plates, and culture medium was replaced after 24 h. Commercially obtained siRNAs contained different target-specific siRNA mixes that were designed to knockdown gene expression.

### Plasmid transfection

We constructed five types of plasmids which were: plasmids that overexpressed ATF4, plasmids that overexpressed CEMIP, plasmids knocking down CEMIP, Bcl-2-ser70 mutant plasmids, and plasmids knocking down PKCα (Vigene Biosciences, Shangdong, China). Recombinant plasmids and empty plasmids were transfected into PC-3 and DU145 cells with Lipofectamine 2000 according to manufacturer protocol. For the sites and sequences of overexpression, knockdown, and mutant plasmids used in this study, please refer to the Supplementary material Tables 4–6 for the sequence.

### Immunofluorescence staining

The mCherry-GFP co-labeled LC3B adenovirus (Beyotime Institute of Biotechnology, Shanghai, China, C3011) was transferred to PCa cells in different treatment groups for 48 h [26]. Autophagy flux were detected in 2D culture, and a total of more than 20 cells under each condition were counted for quantification of autophagy and the images were amplified by 200 times. PKCα antibody (ProteinTech, Chicago, IL, USA) was used to obtain the distribution image of PKCα in PCa cells with stable overexpression of CEMIP. Images were obtained 48 h after transfection of plasmids with partial GFP promoter sequences Beclin1 and Bcl-2 into stable PCa cells with overexpression of CEMIP. In stable PCa cells with overexpression of CEMIP, RFP fluorescent secondary antibody was combined with Bcl-2 and GFP fluorescent secondary antibody with Beclin1, respectively. All data were analyzed via Nikon A1Si Laser Scanning Confocal Microscope (Nikon, Instruments Inc., Japan).

### Co-immunoprecipitation

Subconfluent proliferating cells in 10-cm^2^ dishes were harvested, collected in lysis buffer, left on ice for 30 min, sonicated, and centrifuged at 15,000 rpm for 15 min at 4 °C. Supernatants were collected. Each immunoprecipitation (IP) was carried out using 5-μg antibody and 500-μg protein. The precipitated proteins were collected using protein A + G beads, washed, eluted in boiling Laemmli sample buffer, and subjected to Western blotting. Briefly, 100-μg protein from each group was fractionated on 10% SDS-polyacrylamide gels and transferred to nitrocellulose membranes (Millipore, Bedford, MA, USA). The membranes were then blotted with primary antibodies, followed by the secondary antibody and bathed with enhanced chemiluminescence reagent (Beyotime, P0018s). The primary antibodies used in this study were obtained from ProteinTech (Chicago, IL, USA).

### Chromatin immunoprecipitation (ChIP)

ChIP kit (Beyotime, P2078) was used; the purified DNA was prepared according to the specific operation instructions, and the samples were quantitatively analyzed by quantitative PCR for the combination of ATF4 and CEMIP.

### Luciferase reporter assays

PC-3 and DU145 cells were seeded in 24-well plate (6 × 10^4^ cells per well) 24 h before transfection. The pGL3-basic CEMIP promoter and renilla luciferase reporter vectors (pRL-TK) were co-transfected with ATF4 overexpression plasmid, to determine the combination level of ATF4 and CEMIP. Meanwhile, plasmids of the transcription binding sites of CEMIP (http://jaspar.genereg.net/) were co-transfected with ATF4 overexpression plasmid to determine the specific binding sites of ATF4 and CEMIP. On the other hand, the cells were co-transfected with wild-type/mutant plasmid at the third binding site of CEMIP and the overexpressed ATF4 plasmid to examine the ability of the third binding site to promote transcription. 48 h after transfection the firefly and renilla luciferase activities were measured with Dual-Luciferase® Reporter Assay System (Promega, USA) as described [27].

### Tumor xenografts

PC-3 cells stably labeled by Cy3 and transfected with overexpressed/knockdown CEMIP plasmids or control vector were subcutaneously injected into the upper back of the nude mice (3 × 10^6^, 200 μl). Mice were sacrificed after one month. For animal studies, no blinding was done. The In Vivo FX PRO (BRUKER Corporation, USA) was used to obtain fluorescence images of xenografts in nude mice.

### Statistical analysis

Quantitative data from triplicate experiments were expressed as means values with standard deviation (SD). Statistical analyses were performed by using two-tailed Student’s *t* test or ANOVA, and the Bonferroni corrected Mann-Whitney test was used for post hoc test. The gray value of protein expression was detected by ImageJ software. Statistical analyses were performed by using SPSS (Chicago, IL, USA), and a value of *p* < 0.05 indicated statistical significance.

## Results

### Autophagy is required for PCa cell survival in anoikis conditions

**Fig. 1:**
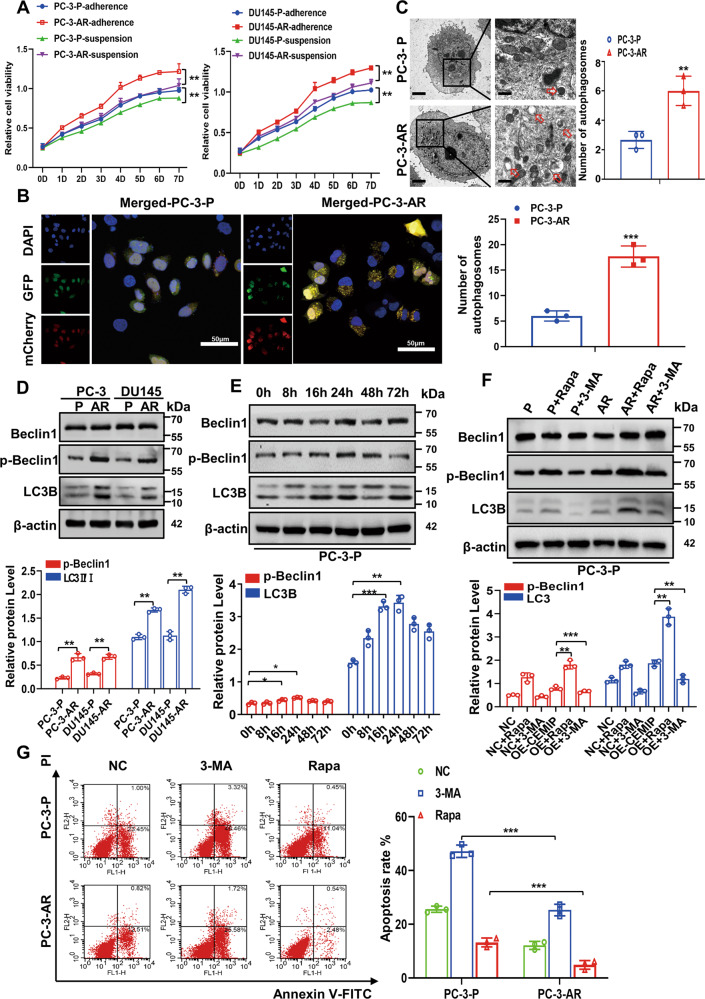
Autophagy is required for survival of PCa cells under anoikis conditions. **A** Cell survival rates in anoikis-resistant (AR) and parental (P) PC-3 and DU145 cells were shown by CCK-8 after continuous suspension and adherent culture for 7 days. **B** Autophagic flux in PC-3-P and PC-3-AR cells was detected by immunofluorescent staining after continuous suspension culture for 48 h, and endogenous LC3B tagged with a tandem fluorescent-mCherry-GFP as a reporter. Autophagy flux were detected in 2D culture, and a total of more than 20 cells under each condition were counted for quantification of autophagy (Scale bar, 50 μm). **C** Transmission electron microscopy manifested that PC-3-AR cells showed more double-membrane autophagosomes compared with PC-3-P cells (Original magnification, ×1000, ×1600, respectively). **D** Western blot analysis of the expression level of autophagy-related proteins (Beclin1, p-Beclin1, LC3BII/LC3BI) in PCa-AR (PC-3-AR、DU145-AR) and PCa-P (PC-3-P、DU145-P) cells. **E** Western blot analysis of the expression level of autophagy-related proteins (Beclin1, p-Beclin1, LC3BII/LC3BI) in PC-3 cells at different suspension time points. **F** Western blot analysis of the expression level of autophagy-related proteins (Beclin1, p-Beclin1, LC3BII/LC3BI) in PC-3-P and PC-3-AR cells after the treatment of Rapa (200 nM) or 3-MA (10 μM). **G** Flow cytometry assay certified the detachment-induced apoptosis rate in parental and anoikis-resistant PC-3 and DU145 cells after the treatment of Rapa (200 nM) or 3-MA (10 μM) in suspension situation for 48 h. (Data are presented as the means ± SD of three independent experiments; *p < 0.05, **p < 0.01, ***p < 0.001).

An anoikis resistance cell model was established using the PC-3 and DU145 cell lines, as described previously [5, 10–12]. The PC-3-AR and DU145-AR cells displayed profound proliferation, migration, invasion, and resistance to apoptosis compared with parental (P) cells (Fig. 1A and Fig. S1A–C). The autophagic flux in the PC-3 and DU145 cells expressing endogenous LC3B, tagged with tandem fluorescent-mCherry-GFP as a reporter, was monitored to further observe the autophagic changes in nonadherent conditions. As shown in Fig. 1B and Fig. S2A, more yellow puncta were evident in the PCa-AR cells than in the parental PCa (PCa-P) cells for 24 h following detachment from the ECM. These results were further verified by TEM, revealing the presence of double-membrane autophagosomes filled with degraded organelles and autolysosomes in the PC-3-AR cells (Fig. 1C). In addition, the autophagy-regulating genes were detected using qRT-PCR and western blotting. Compared with the PCa-P cells, the mRNA in the autophagic genes displayed no significant changes except for the CEMIP in the PC-3-AR and DU145-AR cells (Fig. S2B). However, these cells exhibited a noticeable upregulation in phosphorylated Beclin1 (p-Beclin1) and the LC3BII/LC3BI ratio (Fig. 1D). A western blot analysis was performed on the PCa cells in suspension culture at different times to further investigate the autophagic impact on the anoikis resistance process. The results showed that the LC3BII/LC3BI ratio and p-Beclin1 level increased within 8 h, reaching a peak at 72 h (Fig. 1E), and were distinctly higher after Rapa treatment (200 nM) for 24 h. However, treatment with 3-MA (10 mM) for 24 h reversed this effect (Fig. 1F). The apoptosis rate was assessed using flow cytometry to further determine the role of autophagy during the anoikis resistance process. The apoptosis rate of the PCa-P cells was significantly higher than the PCa-AR cells in suspension conditions, while the apoptosis index was further increased by the 3-MA autophagic inhibitor and partially reversed by the Rapa autophagic inducer (Fig. 1G). These observations indicated that detachment from the ECM triggered protective autophagy, inducing anoikis resistance in the PCa cells.

### CEMIP overexpression is correlated with unfavorable pathological characteristics in PCa

**Fig. 2:**
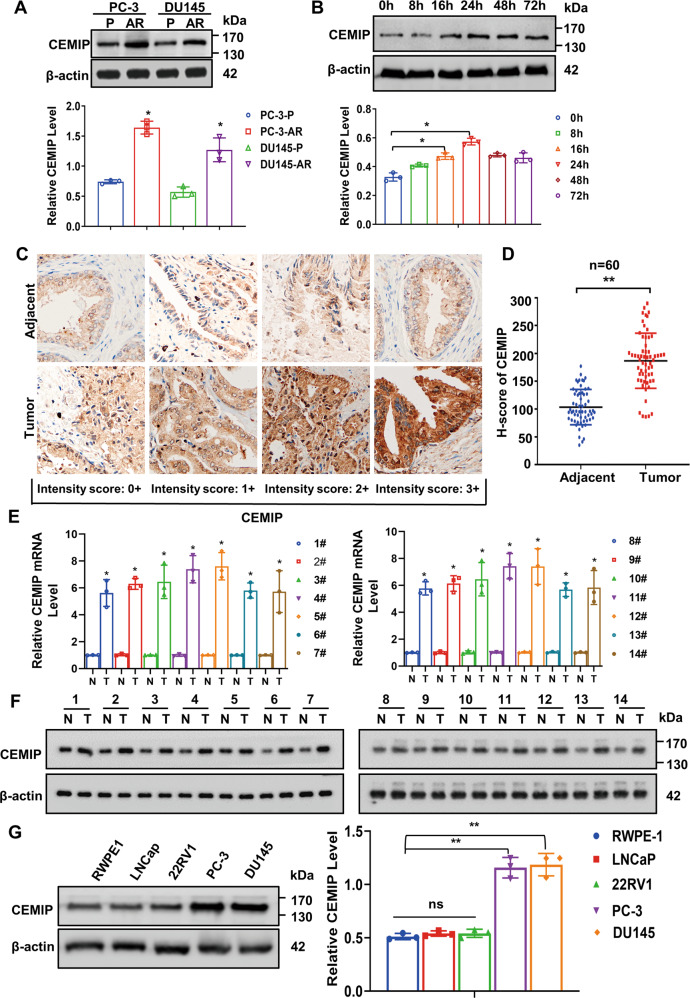
Overexpression of CEMIP is correlated with unfavorable pathological characteristics in PCa. **A** Western blot analysis of the expression level of CEMIP in anoikis-resistant and parental PC-3 and DU145 cells. **B** Western blot analysis of the expression level of CEMIP in PC-3 cells at different suspension time points (0–72 h). **C** Immumohistochemical staining demonstrated the expression level of CEMIP in PCa tissues and corresponding pericarcinous specimens (Original magnification, ×400). **D** The expression of CEMIP in PCa tissues and corresponding adjacent tissues was presented by histochemistry score (*n* = 60, *p < 0.05, **p < 0.01). **E** qRT-PCR analysis of the mRNA level of CEMIP in 14 pairs of human PCa and their adjacent normal tissues. **F** Western blot analysis of the protein level of CEMIP in 14 pairs of PCa tissues and corresponding pericarcinous counterparts. **G** Western blot analysis of the protein expression level of CEMIP in prostatic epithelial cells (RWPE-1) and different PCa cells (LNCaP, 22RV1, PC-3, DU145). (Data are presented as the means ± SD of three independent experiments; *p < 0.05, **p < 0.01).

The western blot assay showed CEMIP overexpression in the PCa-AR cells, compared with the PCa-P cells (Fig. 2A). Further research indicated a significant CEMIP increase in the PCa cells during early suspension culture, which tended to stabilize after 72 h (Fig. 2B). Our previous studies have demonstrated via genome microarray assays that the CEMIP overexpression was substantially higher in the PCa tissues than their pericarcinous counterparts [12]. Immunohistochemical staining was used to determine the CEMIP expression in 60 paired paraffin-embedded PCa and adjacent tissues to further examine the relationship between CEMIP and the pathological and clinical significance of PCa. Consequently, CEMIP expression was found predominantly in the cytoplasm and was distinctly higher in the PCa tissues than in the normal paracancerous tissues (Fig. 2C, D). Furthermore, qRT-PCR analysis (Fig. 2E) and western blotting (Fig. 2F) confirmed that the mRNA and protein levels of CEMIP were significantly elevated in the PCa tissues. Next, the correlation between the CEMIP expression and clinicopathological characteristics was analyzed, showing a positive correlation with the clinical PCa stage (I + II versus III + IV, *P* = 0.0025) (Table 1). The CEMIP expression in several well-known cell lines with varying metastatic potential (RWPE-1, LNCaP, 22RV1, PC-3, and DU145) was determined via western blotting to further investigate the findings of this study in vitro. As expected, the metastatic prostate PC-3 and DU145 cells exhibited the highest protein levels (Fig. 2G) and were consequently selected for the subsequent in vitro study.

**Table 1:** Clinicopathological characteristics of 60 cases of CEMIP expression by immunohistochemistry.

Variable	*n*	CEMIP		*p* value
		High (*n* = 43)	Low (*n* = 17)	
Age (y)				
<65 (median)	26	17	9	0.3955
≥65	34	26	8	
PSA (ng/ml)				
<10	5	2	3	0.1719
10–20	21	15	6	
>20	34	27	7	
Gleason score				
≤6	6	4	2	0.0958
7	32	21	11	
≥8	22	20	2	
Pathological T category				
pT1/2	30	18	12	0.5889
pT3/4	30	21	9	
Lymph node metastasis				
N0	50	36	14	0.7255
N1	10	7	3	
Stage classification				
I–II	23	11	12	0.0025
III–IV	37	32	5	

### CEMIP downregulation attenuates autophagy in PCa-AR cells

**Fig. 3:**
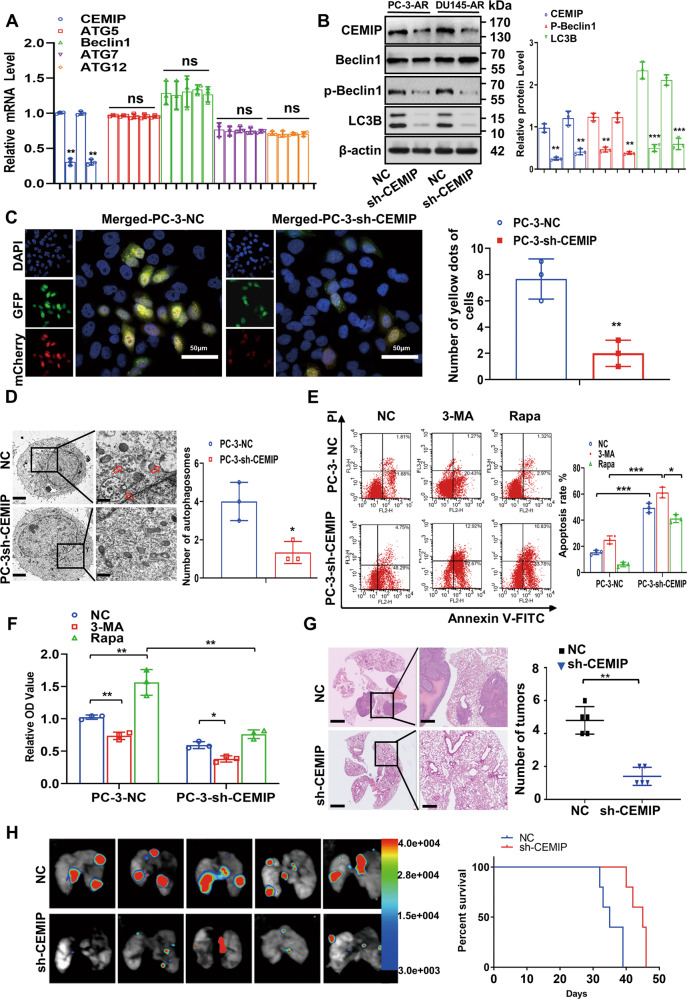
Downregulation of CEMIP attenuates autophagy and cell viability in anoikis-resistant PCa cells. **A** qRT-PCR assay analysis of the mRNA level of Beclin1 and multiple ATGs. **B** Western blot analysis of the Beclin1, p-Beclin1, ATG5, ATG7, ATG9, ATG12, and LC3BII/LC3BI ratio in CEMIP-silenced PC-3-AR and DU145-AR cells. **C** The autophagic flux in CEMIP-silenced PC-3-AR cells and negative control cells was certified by immunofluorescent staining, and endogenous LC3B tagged with a tandem fluorescent-mCherry-GFP as a reporter. Autophagy flux was detected in 2D culture, and a total of more than 20 cells under each condition were counted for quantification of autophagy (Scale bar, 50 μm). **D** Transmission electron microscopy manifested that stable downregulation of CEMIP in PC-3-AR cells attenuated the number of double-membrane autophagosomes (Original magnification, ×1000, ×1600, respectively). **E** The detachment-induced apoptosis in CEMIP-silenced PC-3-AR cells and negative control cells after the treatment of Rapa (200 nM) or 3-MA (10 μM) in suspension situation for 48 h was displayed by flow cytometry assay. **F** Cell viabilities of knockdown CEMIP in PCa-AR cells after the treatment Rapa or 3-MA for 48 h were presented by the CCK-8 assay. PCa-P cells as negative control. **G** H&E staining indicated that knockdown of CEMIP led to decreased number of lung metastatic colonies (*n* = 5 per group, Original magnification, ×10, ×100, respectively). **H** Bioluminescence in vivo imaging showed that knockdown of CEMIP significantly decreased the number of pulmonary metastasis focuses (*n* = 5 per group). Data are presented as the means ± SD of three independent experiments; *p < 0.05, **p < 0.01, ***p < 0.001.

The CEMIP was knocked down in the PC-3-AR and DU145-AR cells using shRNA to assess the CEMIP functionality during autophagy. While no differences were evident in the autophagy markers and multiple ATGs (Fig. 3A and Fig. S3A), the protein levels of p-Beclin1 and LC3BII/LC3BI were reduced in the CEMIP-silenced PCa-AR cells (Fig. 3B). Immunofluorescence staining indicated that the yellow puncta decreased in the CEMIP-silenced PC-3-AR and DU145-AR cells for 24 h after detachment from the ECM (Fig. 3C and Fig. S3B). Furthermore, fewer double-membrane autophagosomes filled with degraded organelles and autolysosomes were found in these cells, indicating that autophagy was inhibited by CEMIP knockdown (Fig. 3D). Apoptosis was then assessed using a flow cytometry assay to further determine the role of autophagy during the suspension process. This suggested that CEMIP downregulation markedly induced PCa-AR cell apoptosis in suspension conditions. As expected, 3-MA promoted apoptosis in the same circumstances, while it was reduced by Rapa (Fig. 3E). Consistent with this, the cell viability determined via CCK-8 was increased by 3-MA and decreased by Rapa (Fig. 3F). Then, the expression of CEMIP and several autophagy-related genes validated the hypothesis of this study that CEMIP downregulation diminished autophagy in the PCa-AR cells (Fig. S3C). Consequently, CEMIP knockdown compromised the aggressive characteristics of PCa-AR cells (Fig. S4) and inhibited in vivo pulmonary metastasis (Fig. 3G, H). A gain-of-function assay was performed to fully understand CEMIP functionality in PCa cells. The stable CEMIP overexpression in the PC-3-P and DU145-P cells significantly enhanced the aggressive characteristics of the PCa cells and promoted in vivo pulmonary metastasis (Fig. S5). While no differences were evident in the autophagy markers and multiple ATGs, the protein levels of p-Beclin1 and LC3BII/LC3BI were elevated in the CEMIP-overexpressed PCa-AR cells (Fig. S6A, B). The results in Fig. S6C–F demonstrated that autophagic activity was significantly promoted in the CEMIP-overexpressed PCa cells, while the cell viability was increased by 3-MA and decreased by Rapa. These findings confirmed that CEMIP induced autophagy and protected ECM-detached PCa cells from anoikis.

### Phosphorylated Bcl-2-ser70 enhances autophagy by promoting the dissociation of the Bcl-2/Beclin1 complex

**Fig. 4:**
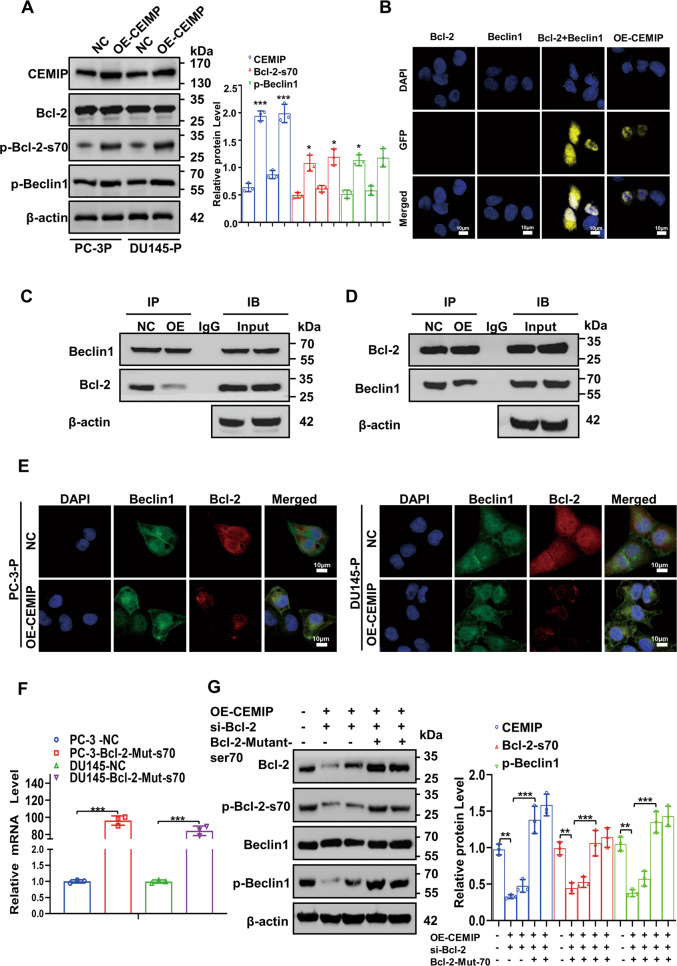
Phosphorylated Bcl-2-ser70 enhances autophagy by promoting dissociation of Bcl-2/Beclin1 complex. **A** Western blot analysis of the expression level of Bcl-2 phosphorylation in serine70 and p-Beclin1 in PC-3 cells stably overexpressing CEMIP. **B** Bimolecular fluorescence complementation assay certified that luminescence occurred only after co-transfection of Bcl-2 and Beclin1 plasmids containing GFP fragments (Scale bar, 10 μm). **C**, **D** The dissociation of Bcl-2/Beclin1 complex in CEMIP-overexpressing PC-3 cells was indicated by Co-IP experiment with Beclin1 antibody (C) and Bcl-2 antibody (**D**). **E** Immunofluorescence staining revealed the dissociation of the Bcl-2/Beclin1 complex in CEMIP-overexpressing PC-3 cells. Nuclei were stained blue by DAPI, Beclin1 were stained by GFP-labeled antibody, Bcl-2 were stained by RFP-labeled antibody (Scale bar, 10 μm). **F** The efficiency of Bcl-2-mutant plasmids was certified by qRT-PCR after transfection for 48 h in PC-3 and DU145 cells. **G** Rescue western blot assay comprehensively demonstrated that overexpression of CEMIP enhanced autophagy by phosphorylating Bcl-2-ser70. Data are presented as the means ± SD of three independent experiments; *p < 0.05, **p < 0.01, ***p < 0.001.

The Bcl-2 phosphorylation level was significantly increased in the PCa cells exhibiting stable CEMIP overexpression, while the activity of the autophagy-related gene, p-Beclin1, was higher (Fig. 4A). To identify the molecular mechanisms underlying Bcl-2 phosphorylation in CEMIP-promoted autophagy, GFP-labeled fragment plasmids carrying Bcl-2 and Beclin1 were constructed for the bimolecular fluorescence complementation assay. The results indicated that luminescence only occurred after the co-transfection of the Bcl-2 and Beclin1 plasmids containing GFP fragments (Fig. 4B). However, CEMIP overexpression promoted Bcl-2 and Beclin1 dissociation and impaired the binding of the Bcl-2/Beclin1 complex detected via the Co-IP and immunofluorescence assay (Fig. 4C, E). Next, the efficiency of the Bcl-2-mutant plasmids in the PC-3 and DU145 cells was verified 48 h after transfection using qRT-PCR (Fig. 4F). Rescue experiments further confirmed that CEMIP overexpression in the PCa-P cells enhanced autophagy via Bcl-2-ser70 phosphorylation (Fig. 4G). Therefore, CEMIP induced Bcl-2-ser70 phosphorylation, promoted the dissociation of the Bcl-2/Beclin1 complex, and mediated protective autophagy in the PCa cells.

### PKCα membrane transposition leads to CEMIP mediation of Bcl-2 phosphorylation at ser70

**Fig. 5:**
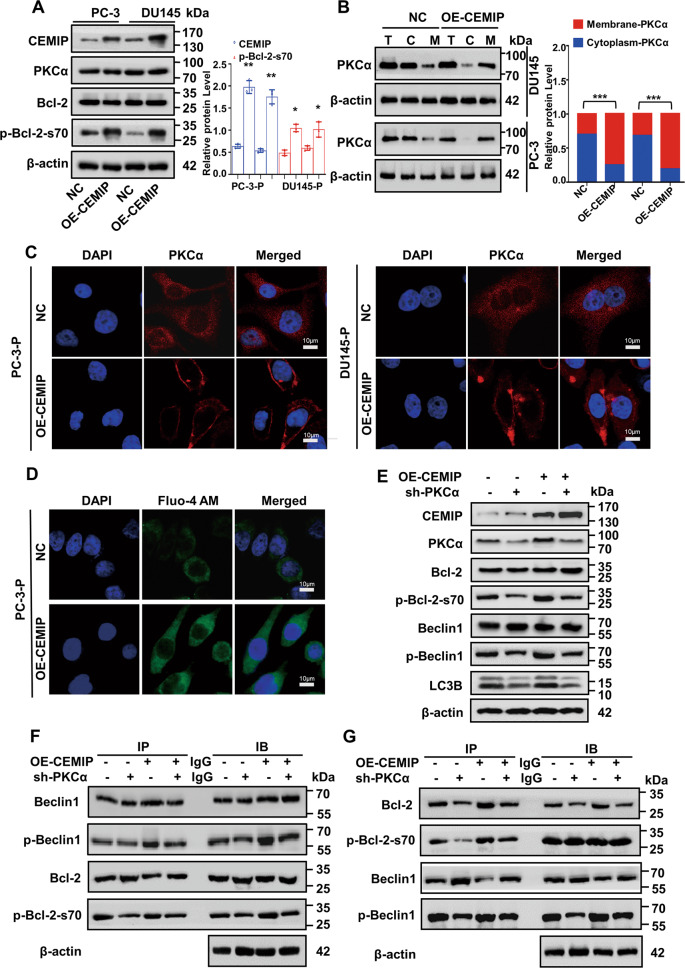
CEMIP phosphorylates ser70 site of Bcl-2 in PCa cells by promoting membrane transposition of PKCα. **A** Western blot assay demonstrated the proteins level of phosphorylated Bcl-2-ser70 and PKCα in CEMIP-overexpressing PC-3-P and DU145-P cells. **B** The membrane translocation of PKCα in overexpressing CEMIP PC-3-P and DU145-P cells was presented by Membrane and Cytosol Protein Extraction Kit. **C** Immunofluorescence staining certified the membrane translocation in PKCα. Nuclei were stained blue by DAPI, PKCα was stained by RFP-labeled antibody (Scale bar, 10μm). **D** The intracellular cytoplasm calcium ions level in CEMIP-overexpressing PC-3-P cells were detected by immunofluorescence staining. Nuclei were stained blue by DAPI, calcium ions were stained green by Fluo-4 AM (Scale bar, 10 μm). **E** Rescue western blot assay demonstrated the reversion efficiency of knocking down PKCα after CEMIP overexpression. **F**, **G** The phosphorylation of Bcl-2-ser70 and dissociation of Bcl-2/Beclin1 complex were verified by rescue CoIP assay after the co-transfection of overexpressing CEMIP plasmids and knockdown PKCα plasmids for 72 h. Data are presented as the means ± SD of three independent experiments; *p < 0.05, **p < 0.01, ***p < 0.001.

Although the phosphorylation level of Bcl-2 increased, followed by stable CEMIP overexpression, the latter was a classic protein kinase. Recently, CEMIP was found to promote PKCα membrane transposition, enhancing its activity in breast cancer cells [19]. Here, the total amount of PKCα protein remained unchanged, but the membrane distribution increased in the PC-3 and DU145 cells, exhibiting stable CEMIP expression (Fig. 5A, B). In addition, immunofluorescence staining confirmed the PKCα translocation from the cytosol to the plasma membrane in CEMIP-overexpressed PCa-P cells (Fig. 5C). More specifically, CEMIP overexpression increased the intracellular calciumion level (Fig. 5D and Fig. S7A, B), which was consistent with previous reports [16]. To determine whether PKCα was required for CEMIP-mediated autophagy, the PCKα was silenced with shRNA in the CEMIP-expressed PCa cells (Fig. S7C). A rescue western blot assay showed that the PKCα partially reversed the bio-effects caused by CEMIP overexpression (Fig. 5E and Fig. S7D). A rescue Co-IP experiment was performed to validate these findings, showing that silencing the PKCα in the CEMIP-overexpressed PCa-P cells reduced Bcl-2-ser70 phosphorylation, subsequently promoting the dissociation of the Bcl-2/Beclin1 complex (Fig. 5F, G). These findings demonstrated that CEMIP-mediated PKCα membrane translocation was responsible for Bcl-2-ser70 phosphorylation and subsequent autophagy.

### ATF4 acts as a co-transcription factor to promote CEMIP transcription in PCa-AR cells

**Fig. 6:**
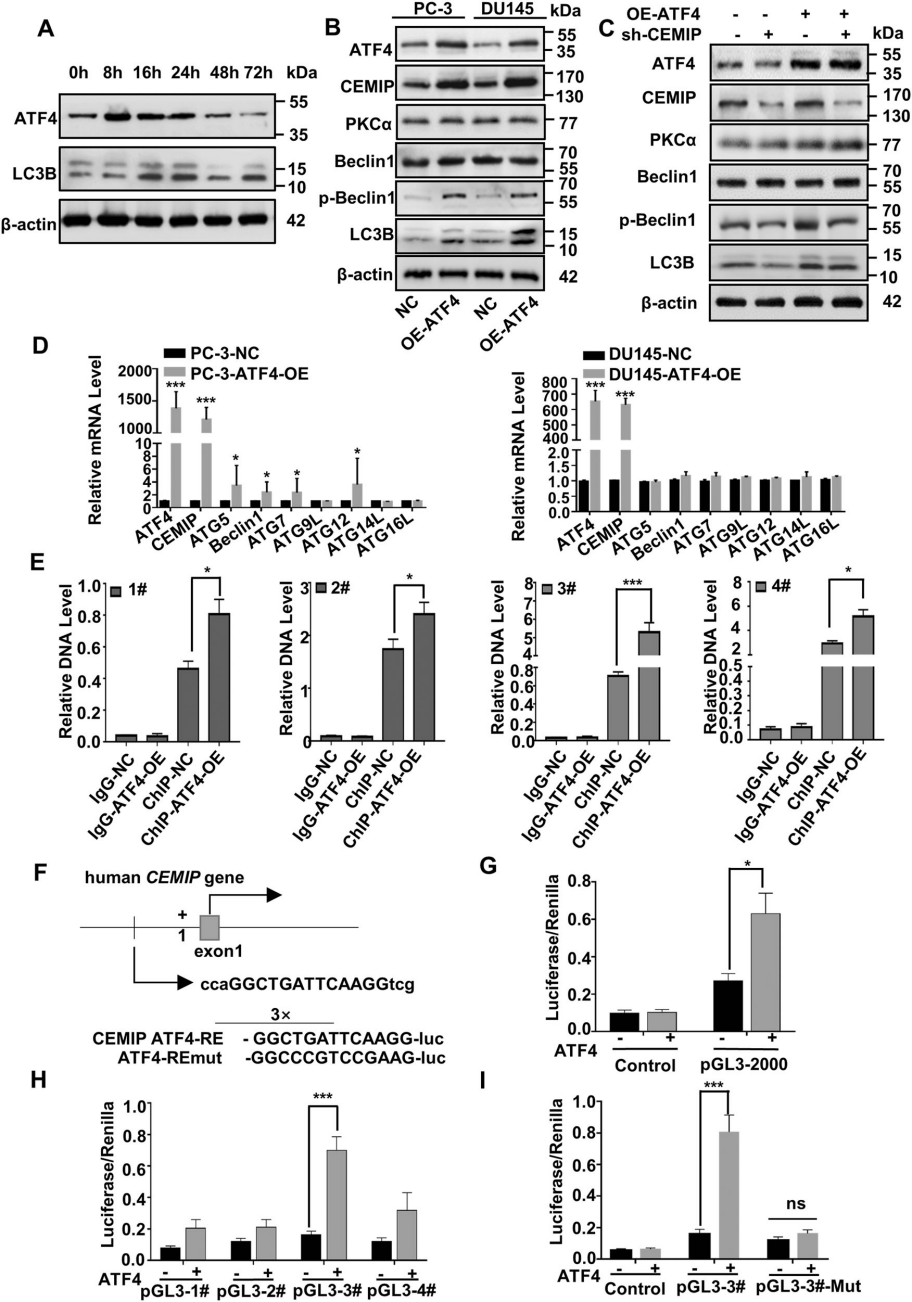
ATF4 acts as co-transcription factor to promote transcription of CEMIP in anoikis-resistant PCa cells. **A** Western blot analysis of the proteins level of ATF4 and LC3BII/LC3BI in PCa cells at different suspension time points. **B** The protein level of CEMIP, p-Beclin1 and LC3BII/LC3BI in ATF4-overexpressed PC-3-P and DU145-P cells were presented by western blot assay. **C** Rescue western blot analysis of the reversion efficiency in autophagy of knocking down CEMIP after the stable overexpression of ATF4 in PC-3-P and DU145-P cells. **D** qRT-PCR analysis of the transfection efficiency of ATF4 after for 48 h in PC-3 and DU145 cells. **E** ChIP assay was performed to find the potential transcription binding sites of ATF4 for CEMIP. **F** Specificly designed binding sites of ATF4 and sequences on CEMIP promoter. **G** The luciferase activity of CEMIP 3′UTR and promoter after transfection with ATF4 overexpression plasmids in PCa-P cells. **H** The luciferase activities of the four transcription binding sites of CEMIP 3′UTR after transfection with ATF4 overexpression plasmids in PCa cells. **I** The luciferase activity of the binding site 3 of CEMIP 3′UTR after cotransfection with ATF4 overexpression plasmids and binding site 3 mutant CEMIP plasmids in PCa cells. Renilla as internal reference. Data are presented as the means ± SD of three independent experiments; *p < 0.05, **p < 0.001.

ATF4 reportedly activates protective autophagy and antioxidant responses while protecting cells from anoikis [9, 18]. Western blot analysis showed that the ATF4 increased significantly in the PCa cells in a time-dependent manner as early as 8 h after suspension culture, peaking between 8–24 h of continuous suspension (Fig. 6A and Fig. S8A), which was earlier than CEMIP (Fig. 2B). Therefore, the suspension time point was set to 72 h of continuous suspension. In addition, the western blot assay confirmed that the expression levels of ATF4 and CEMIP in typical PCa cells (LNCaP, 22RV1, PC-3, and DU145) were significantly upregulated compared with prostate epithelial cells (RWPE-1), especially in the PC-3 and DU145 (Fig. S8B). The ATF4 overexpression plasmid was transfected into the PC-3-P and DU145-P cells, confirming CEMIP overexpression and the presence of the autophagy-related proteins (p-Beclin1 and LC3BII/LC3BI) to explore the impact of ATF4 in CEMIP-induced PCa-AR, as shown in Fig. 6B and Fig. S8C. A rescue experiment was performed by co-transfecting PCa-P cells with ATF4-overexpressed and CEMIP-knockdown plasmids to further investigate the regulatory effect of ATF4 on upstream CEMIP. As expected, CEMIP downregulation partially reversed the bio-effect caused by ATF4 overexpression (Fig. 6C and Fig. S8D). In addition, the ATF4 mRNA expression in the PC-3-AR and DU145-AR cells was examined using qRT-PCR (Fig. S2A). ATF4 overexpression increased the CEMIP transcription without altering autophagy-related molecule transcription (Fig. 6D).

Finally, a ChIP assay was performed to further reveal the mechanism underlying ATF4-mediated CEMIP transcription. JASPAR (http://jaspar.genereg.net/) indicated four potential transcription binding sites for CEMIP, for which this study designed specific PCR primers. The gene expression at the different CEMIP transcription sites was determined, showing that the binding site 3 level was the highest (Fig. 6E). Next, a dual-luciferase reporter assay was conducted to identify the direct binding between CEMIP and ATF4. Reporter plasmids carrying CEMIP promoters were constructed for all the binding sites, as well as a mutant plasmid for binding site 3 (specific design sites and sequences are shown in Fig. 6F). The luciferase reporter activity was about twice as high in the experimental group than in the control group (Fig. 6G). Additionally, the luciferase intensity increased in all groups with CEMIP promoters, especially at binding site 3, which was three times higher than in the control group (Fig. 6H). ATF4 overexpression profoundly enhanced CEMIP 3′UTR luciferase reporter activity during the next phase, while no substantial alternation was evident in the mutant plasmid of binding site 3 (Fig. 6I). These findings demonstrate that ATF4 may bind to CEMIP 3′UTR directly, most likely, at transcription binding site 3, consequently promoting the CEMIP transcription levels.

## Discussion

Anoikis resistance acquisition by cancer cells upon detachment from the ECM facilitates their survival in vascular and lymphatic vessels as well as migration to secondary sites, promoting metastasis. Anoikis tolerance is also responsible for the treatment failure of various types of cancer. Therefore, anoikis resistance is regarded as a hallmark of metastatic cancer cells and is essential for tumor progression, including in PCa cells [28]. Although many studies are available regarding the mechanisms underlying anoikis resistance, how PCa cells escape anoikis remains unclear. CEMIP was previously screened as a potential molecular target via genome microarray analysis, demonstrating that it promoted anoikis resistance in PCa cells by regulating metabolic reprogramming [12].

This study confirmed that the CEMIP expression was substantially higher in late-stage PCa tissue than in pericarcinous tissue. Furthermore, CEMIP is vital for the proliferation, migration, and invasion of cancer cells [29, 30]. However, minimal studies are available regarding the context-specific functionality of CEMIP in cancer cell survival in suspension conditions.

Autophagy is a lysosome-dependent process in which enzymatic degradation and the recycling of cytosolic components occur following cell exposure to stressful conditions [3, 5]. The impact of autophagy in cancer progression is complicated since autophagy can be twofold (i.e., a pro-survival or pro-death agent depending on the context and the stimuli). Recent studies revealed that autophagy prevented anoikis when exposed to the stress of ECM detachment. For example, attachment-induced autophagy loss significantly increased anoikis resistance via Spi-B transcription factor activation in lung cancer cells [31]. Another study confirmed that astrocyte-elevated gene 1 increased after being isolated from the ECM of hepatocellular carcinoma cells and could induce anoikis resistance by activating autophagy [32]. Furthermore, an extracellular acidic environment induced autophagy by downregulating MiR-3663-3p and promoted anoikis resistance in hepatocellular carcinoma cells [33]. The present study indicated that CEMIP-mediated protective autophagy was required for PCa cell anoikis resistance. ATF4 directly bound to CEMIP 3′UTR, increasing the CEMIP transcription levels. Furthermore, the plasma membrane transposition of PKCα promoted Bcl-2 phosphorylation at the ser70 site, which was essential for CEMIP-mediated autophagy. Silencing PKCα reversed the CEMIP induced autophagy in suspension conditions. Elevated Bcl-2-ser70 phosphorylation promoted the dissociation of the Bcl-2/Beclin1 complex, enhancing protective autophagy and encouraging PCa cell anoikis resistance (Fig. 4).

Beclin1 is crucial in initiating autophagolysosome formation, which functions via phosphorylation [34, 35]. This study showed that CEMIP-promoted Beclin1 phosphorylation in PCa cells (Fig. S6B and Fig. S6F). Beclin1 was first discovered due to the anti-apoptotic protein, Bcl-2, which inhibited autophagy by interacting with the Bcl-2 homology 3 domain of Beclin1 [36]. The results illustrated that the dissociation of the Bcl-2/Beclin1 complex enhanced autophagy and promoted cell survival [37–39], while CEMIP overexpression significantly increased Bcl-2 during ser70 phosphorylation (Fig. 5A). Moreover, the dissociation of the Bcl-2/Beclin1 complex increased, triggering significant autophagic activation. Contrarily, CEMIP suppression decreased the basal level of autophagy in the PCa-AR cells by attenuating Bcl-2-ser70 phosphorylation and Bcl-2 and Beclin1 disruption. For the first time, this study reports that CEMIP promotes Beclin1-mediated autophagy via Bcl-2 in PCa cell phosphorylation.

CEMIP is unlikely to phosphorylate Bcl-2 directly as a phosphorylated kinase. Therefore, it is hypothesized that CEMIP may regulate the activity of other phosphorylated kinases since it is responsible for the positive regulation of PKCα activity [18]. PKCα belongs to the serine/threonine kinase family and regulates anoikis resistance in human cancer cells [40, 41]. The calcium leakage from the ER, mediated by CEMIP, promoted PKCα transposition from the cytosolic compartment to the plasma membranes in MCF-7 cells [18]. This type of membrane transposition is essential for PKCα activation and functionality [42, 43]. As expected, PKCα localization occurred predominantly in the cell membrane and increased dramatically in the CEMIP-overexpressed PCa-P cells compared with the control cells. Furthermore, silencing the PKCα in the CEMIP-overexpressed PCa-P cells reversed the CEMIP-mediated phosphorylation of Bcl-2-ser70 and autophagy (Fig. 5d). This study indicated that CEMIP-promoted PKCα translocation to the membrane, while the activated PCKα phosphorylated Bcl-2 at ser70, mediating protective autophagy in the ECM-detached PCa cells.

ATF4 is a vital transcription factor in the ER stress [9, 17, 18] caused by the detachment of mammalian epithelial cells from the ECM [1, 44], transcriptionally activating multiple downstream genes that promote adaption and cell survival when exposed to anoikis [45, 46]. In addition, previous research confirmed that tumor cells could be protected from anoikis and contribute to tumor metastasis by activating the coordination program of autophagy and antioxidant response when they were stressed or separated from the ECM [47]. Another study indicated that the ATF4 pathway was activated, and the DDIT4 was upregulated to restrict mTOR and promote autophagy [48] after inhibiting glutamine decomposition in colorectal tumor cells. Therefore, ATF4 and CEMIP were activated in a time-dependent manner upon PCa cell detachment, while both the activation and peak time of ATF4 occurred earlier than CEMIP (Fig. 2B and Fig. 6A). The results further confirmed that ATF4 bonded to transcription binding site 3 of CEMIP, promoting the CEMIP transcription levels in suspended PCa cells (Fig. S6H, I). This evidence links ATF4 activation to CEMIP overexpression during PCa cell anoikis resistance acquisition.

**Fig. 7:**
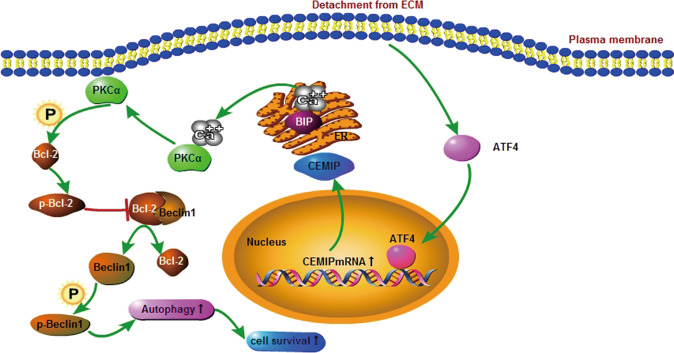
Schematic diagram indicates the CEMIP regulating pathway in PCa cells. Under detachment conditions, ATF4 triggered CEMIP transcription and enhanced PKCα membrane translocation, which regulated the serine70 phosphorylation of Bcl-2, while the subsequent dissociation of the Bcl-2/Beclin1 complex led to autophagy and cell survival.

This study reveals the crucial role of CEMIP as the downstream target gene of ATF4. ATF4/CEMIP-promoted PKCα/Bcl-2-regulated protective autophagy during the anoikis resistance of castration-resistant PCa cells by enhancing the disruption of the Bcl-2/Beclin1 complex (Fig. 7). The results of this research pave the way for additional investigation regarding therapeutic strategies for advanced PCa treatment.

### Reporting summary

Further information on research design is available in the Nature Research Reporting Summary linked to this article.

## Supplementary information

Supplementary material file

Reporting Summary

## Data Availability

The datasets used and/or analyzed during the current study are available from the corresponding author on reasonable request.
