# Supplementary material for: ATF4/CEMIP/PKCα promotes anoikis resistance by enhancing protective autophagy in prostate cancer cells
Source: Cell Death Dis. 2022 Jan 10;13(1):46. doi: 10.1038/s41419-021-04494-x (PMC8748688; doi:10.1038/s41419-021-04494-x)
Supplement: Supplementary file 1 — Supplementary material file [file 41419_2021_4494_MOESM1_ESM.pdf]

Fig. S1

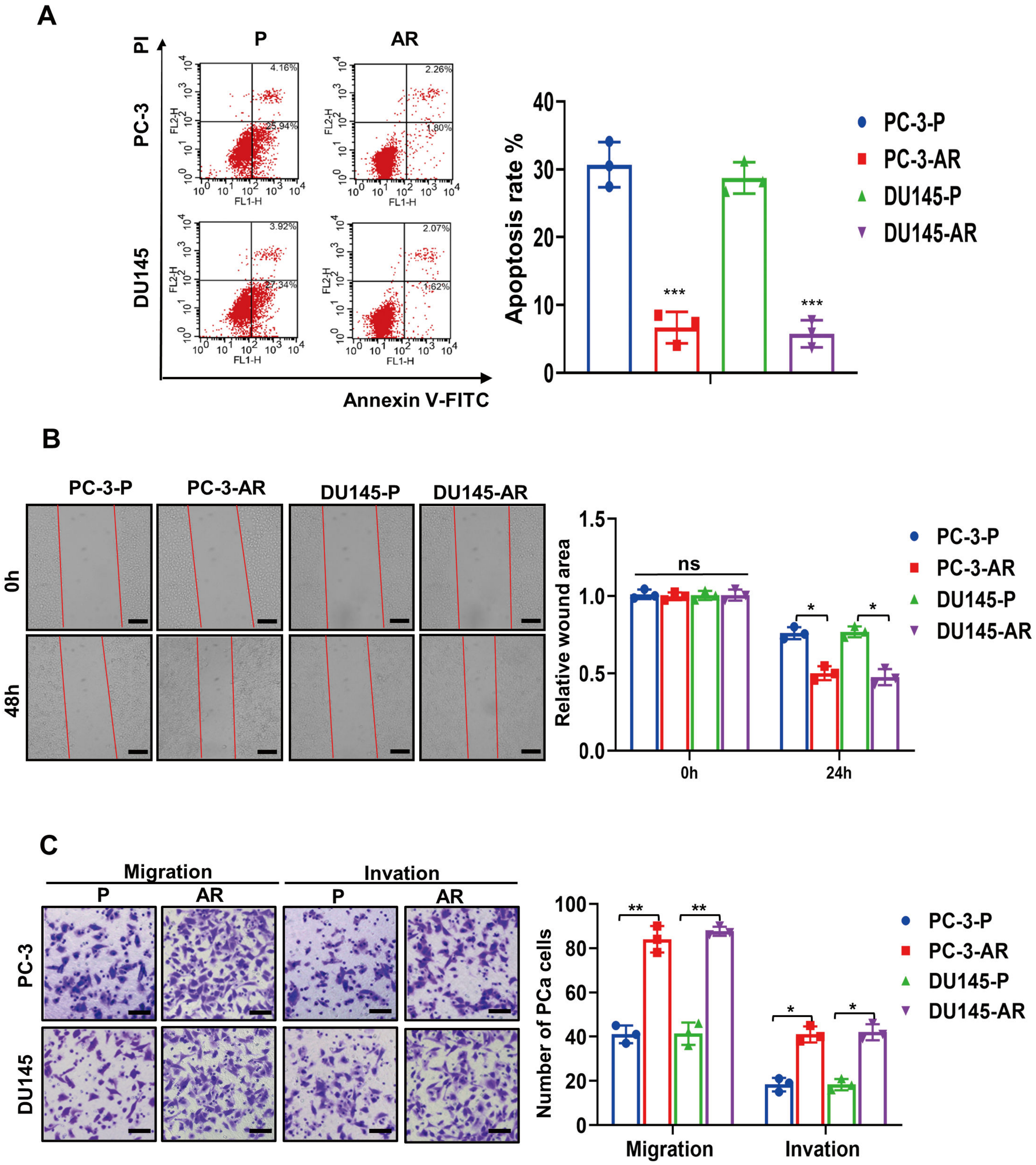
A
P
AR
PI
PC-3
DU145
4.16%
25.94%
2.26%
1.80%
3.92%
27.34%
2.07%
1.62%
FL2-H
FL1-H
Annexin V-FITC
Apoptosis rate %
***
***
PC-3-P
PC-3-AR
DU145-P
DU145-AR
B
PC-3-P
PC-3-AR
DU145-P
DU145-AR
0h
48h
Relative wound area
ns
*
*
0h
24h
C
Migration
Invation
P
AR
P
AR
PC-3
DU145
Number of PCa cells
**
**
*
*
Migration
Invation

Fig. S2

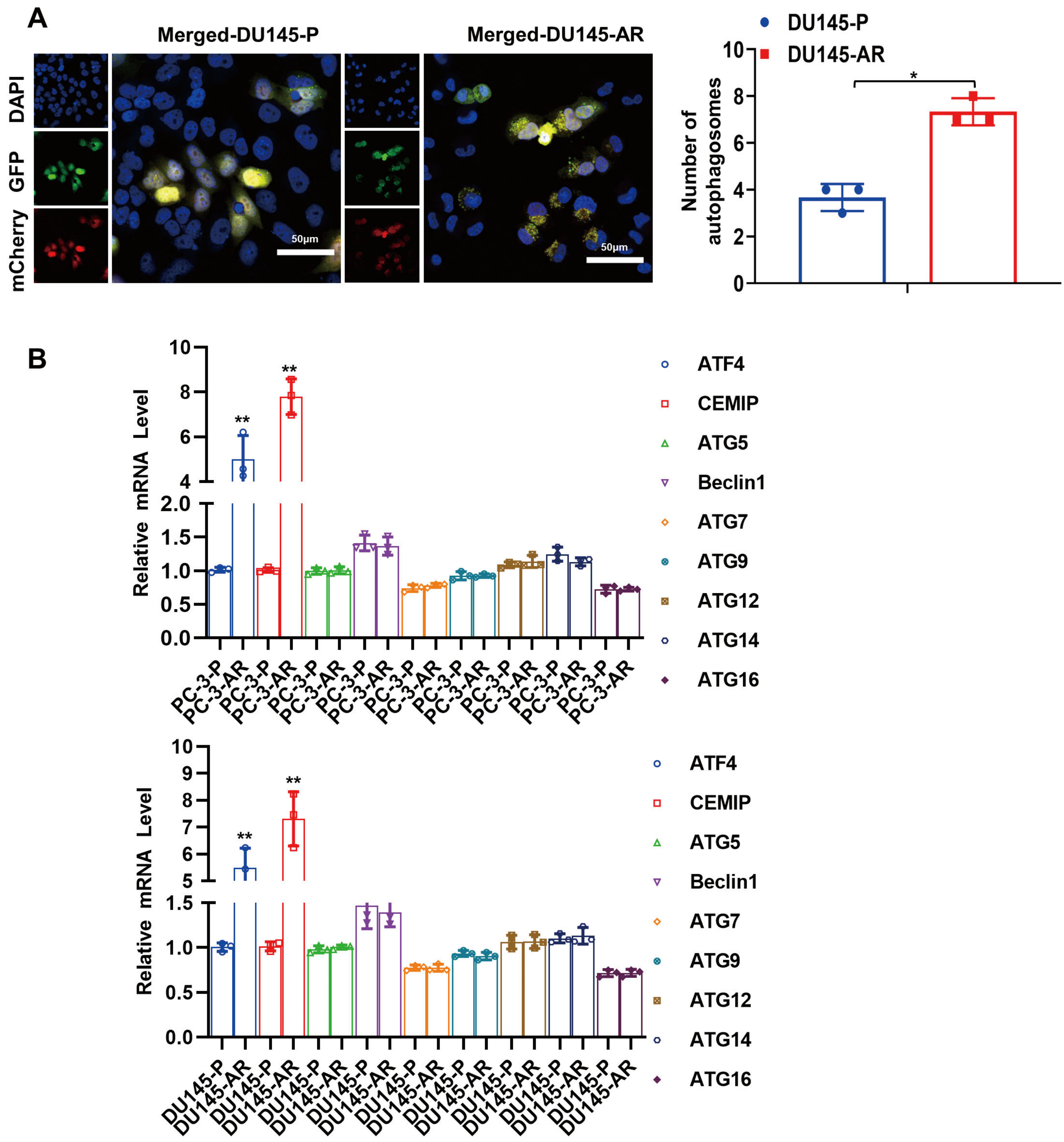
A
Merged-DU145-P
Merged-DU145-AR
DAPI
GFP
mCherry
50μm
50μm
DU145-P
DU145-AR
Number of autophagosomes
*
B
Relative mRNA Level
**
**
PC-3-P
PC-3-AR
ATF4
CEMIP
ATG5
Beclin1
ATG7
ATG9
ATG12
ATG14
ATG16
DU145-P
DU145-AR

Fig. S3

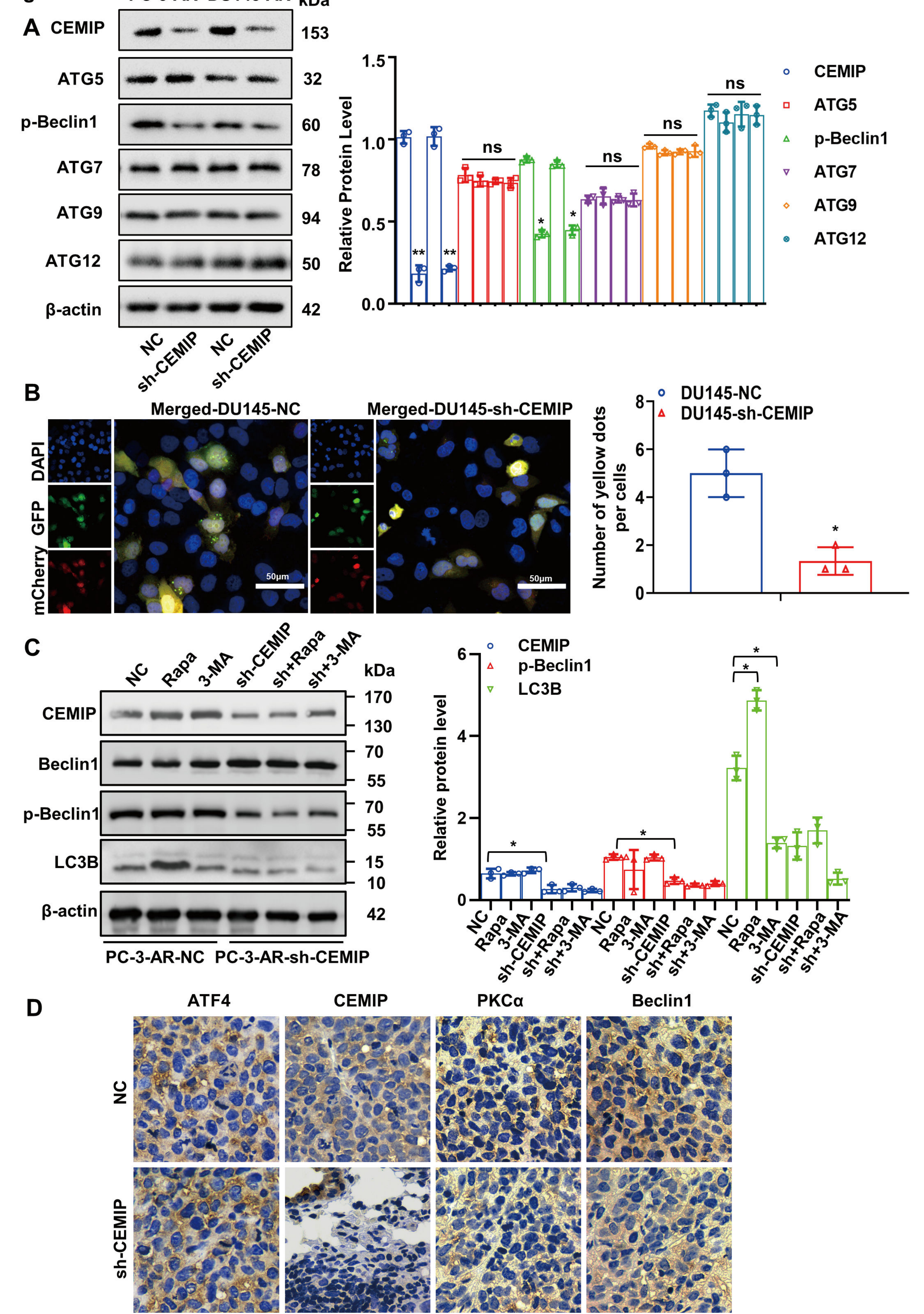

A
PC-3-AR DU145-AR kDa
CEMIP 153
ATG5 32
p-Beclin1 60
ATG7 78
ATG9 94
ATG12 50
β-actin 42
NC sh-CEMIP NC sh-CEMIP
Relative Protein Level
CEMIP
ATG5
p-Beclin1
ATG7
ATG9
ATG12
ns
B
Merged-DU145-NC
Merged-DU145-sh-CEMIP
DAPI
GFP
mCherry
50μm
DU145-NC
DU145-sh-CEMIP
Number of yellow dots per cells
C
NC Rapa 3-MA sh-CEMIP sh+Rapa sh+3-MA
kDa
CEMIP
Beclin1
p-Beclin1
LC3B
β-actin
PC-3-AR-NC PC-3-AR-sh-CEMIP
CEMIP
p-Beclin1
LC3B
Relative protein level
D
ATF4 CEMIP PKCα Beclin1
NC
sh-CEMIP

Fig. S4

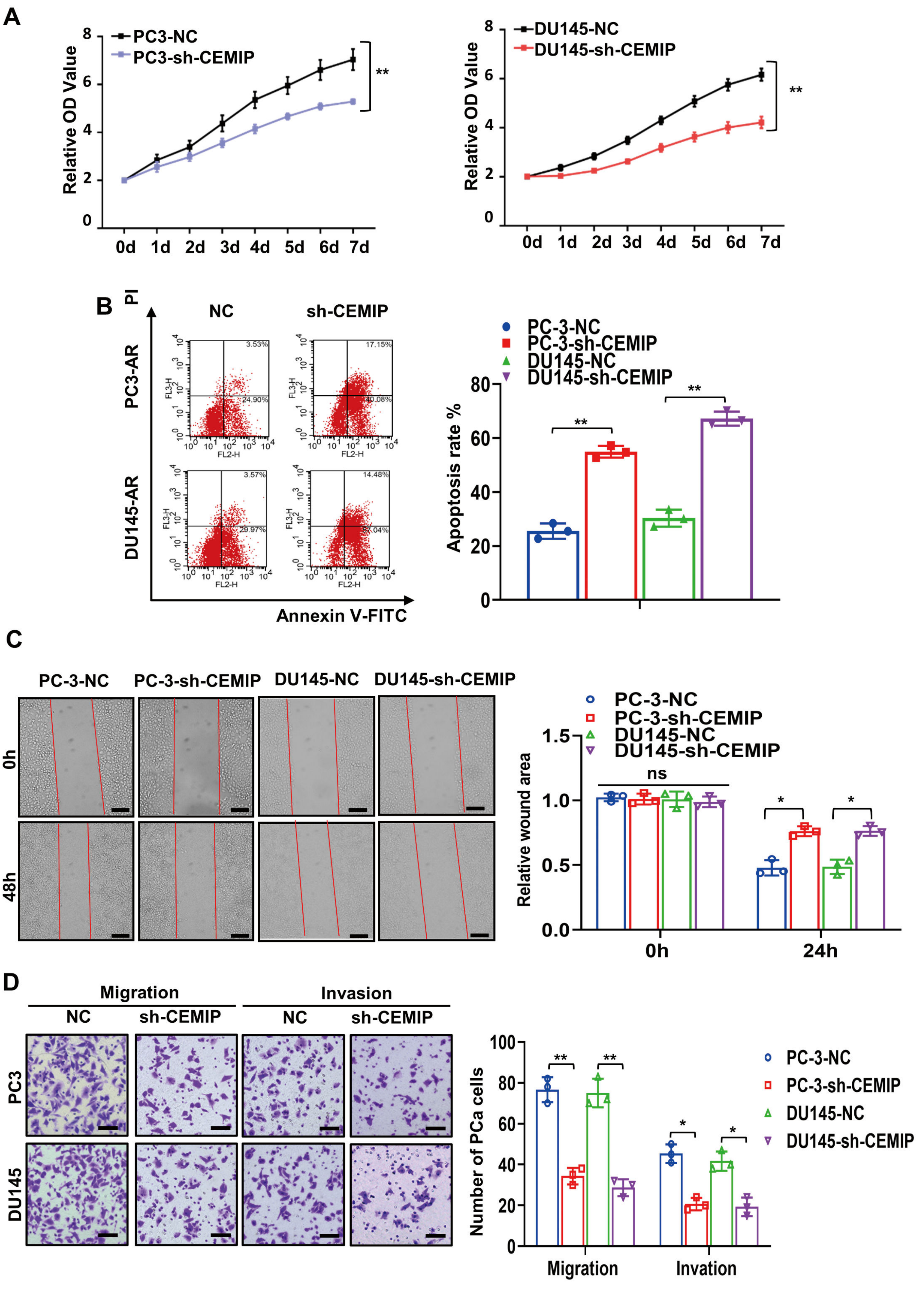
A
PC3-NC
PC3-sh-CEMIP
Relative OD Value
0d 1d 2d 3d 4d 5d 6d 7d
**
DU145-NC
DU145-sh-CEMIP
B
PI
NC
sh-CEMIP
PC3-AR
DU145-AR
Annexin V-FITC
PC-3-NC
PC-3-sh-CEMIP
DU145-NC
DU145-sh-CEMIP
Apoptosis rate %
C
0h
48h
Relative wound area
ns
24h
D
Migration
Invasion
PC3
DU145
Number of PCa cells
Invation

Fig. S5

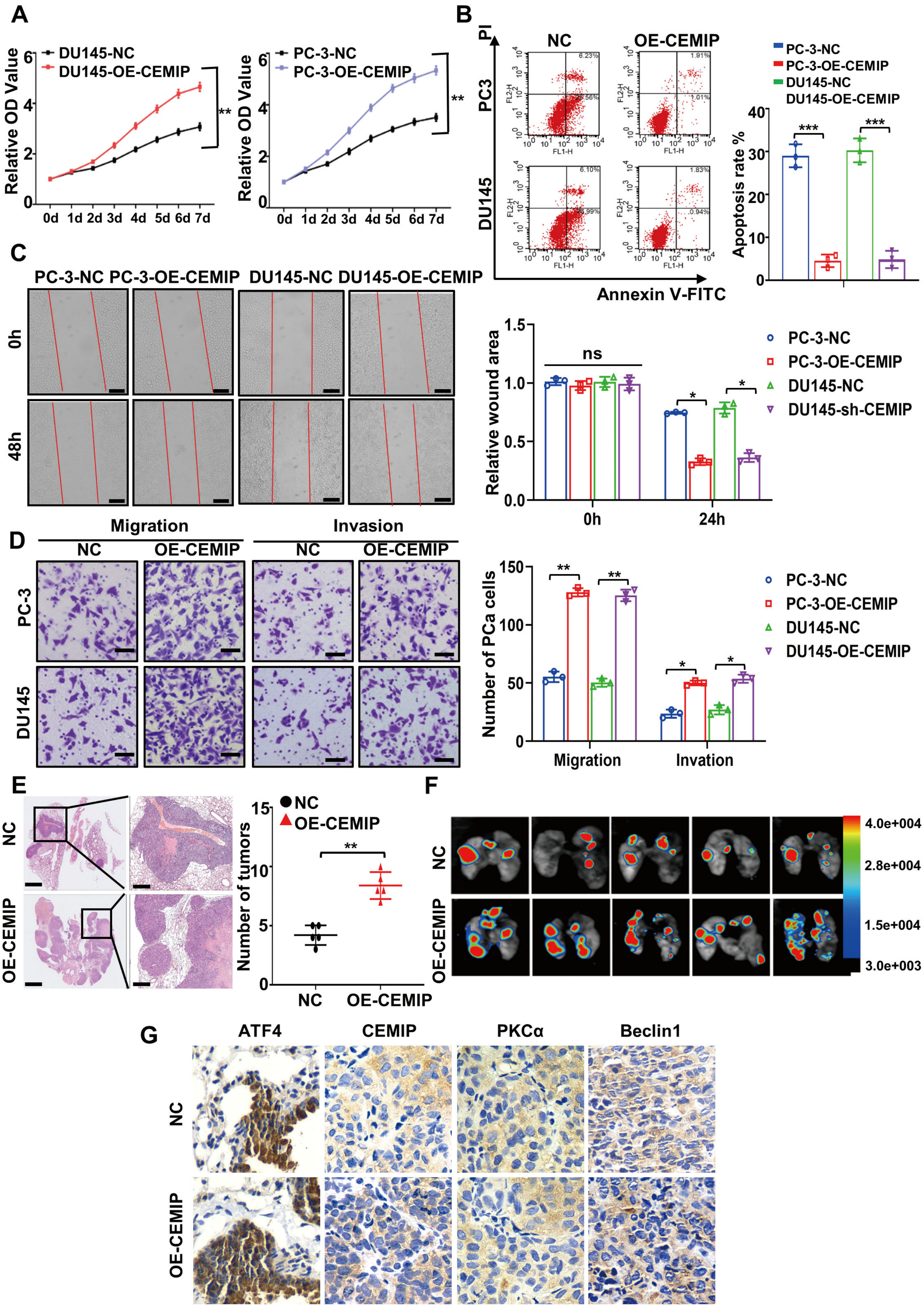
A
DU145-NC
DU145-OE-CEMIP
Relative OD Value
PC-3-NC
PC-3-OE-CEMIP
0d 1d 2d 3d 4d 5d 6d 7d
B
PI
NC
OE-CEMIP
PC3
DU145
Annexin V-FITC
PC-3-NC
PC-3-OE-CEMIP
DU145-NC
DU145-OE-CEMIP
Apoptosis rate %
C
PC-3-NC PC-3-OE-CEMIP DU145-NC DU145-OE-CEMIP
0h
48h
Relative wound area
ns
DU145-sh-CEMIP
24h
D
Migration
Invasion
PC-3
Number of PCa cells
Invation
E
OE-CEMIP
Number of tumors
F
4.0e+004
2.8e+004
1.5e+004
3.0e+003
G
ATF4
CEMIP
PKCα
Beclin1

Fig. S6

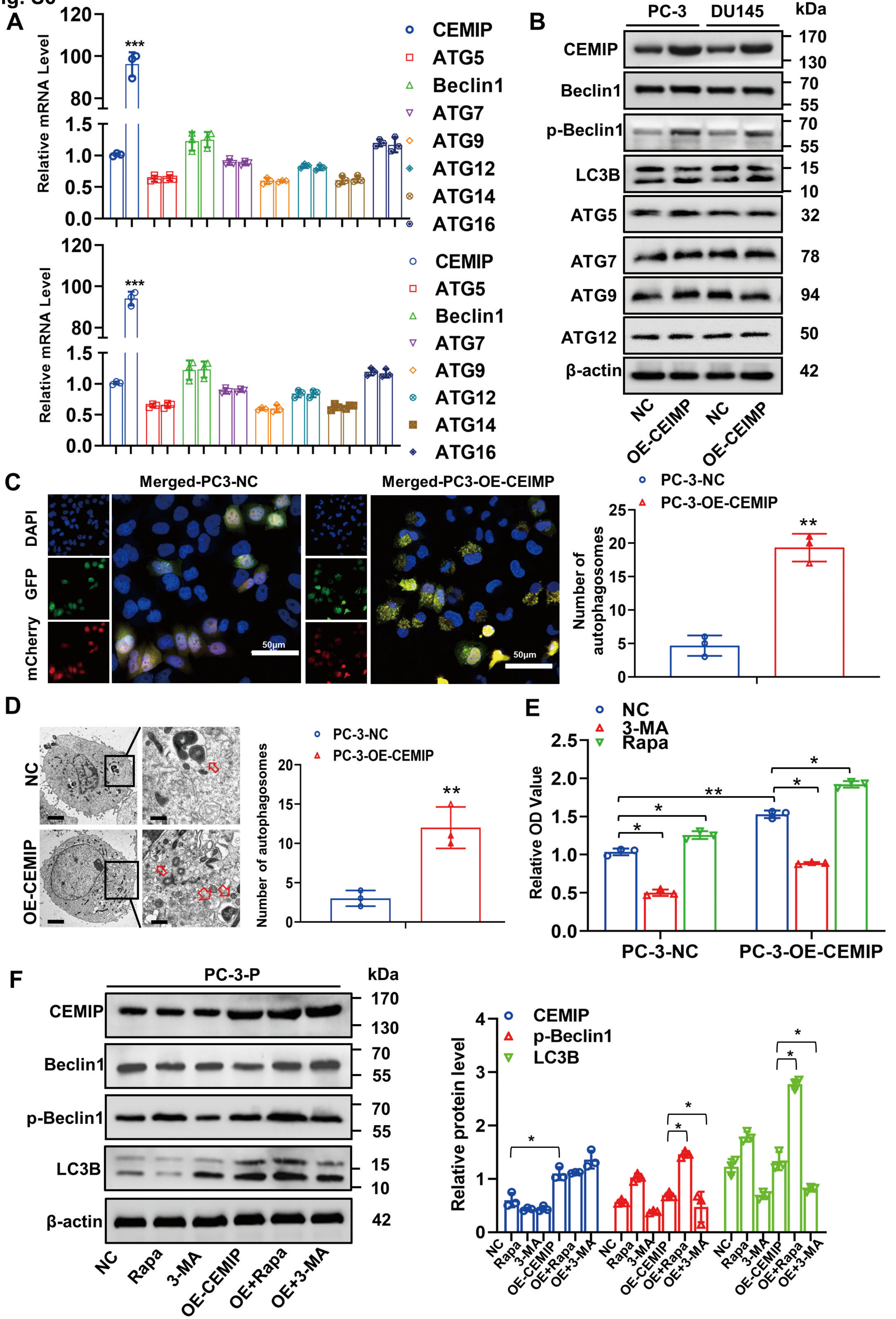
A
Relative mRNA Level
***
CEMIP
ATG5
Beclin1
ATG7
ATG9
ATG12
ATG14
ATG16
B
PC-3
DU145
kDa
CEMIP
Beclin1
p-Beclin1
LC3B
ATG5
ATG7
ATG9
ATG12
β-actin
170
130
70
55
15
10
32
78
94
50
42
NC
OE-CEIMP
C
Merged-PC3-NC
Merged-PC3-OE-CEIMP
DAPI
GFP
mCherry
50μm
PC-3-NC
PC-3-OE-CEMIP
Number of autophagosomes
**
D
NC
OE-CEMIP
E
NC
3-MA
Rapa
Relative OD Value
PC-3-NC
PC-3-OE-CEMIP
F
PC-3-P
Rapa
3-MA
OE+Rapa
OE+3-MA
CEMIP
p-Beclin1
LC3B
Relative protein level
*

Fig. S7

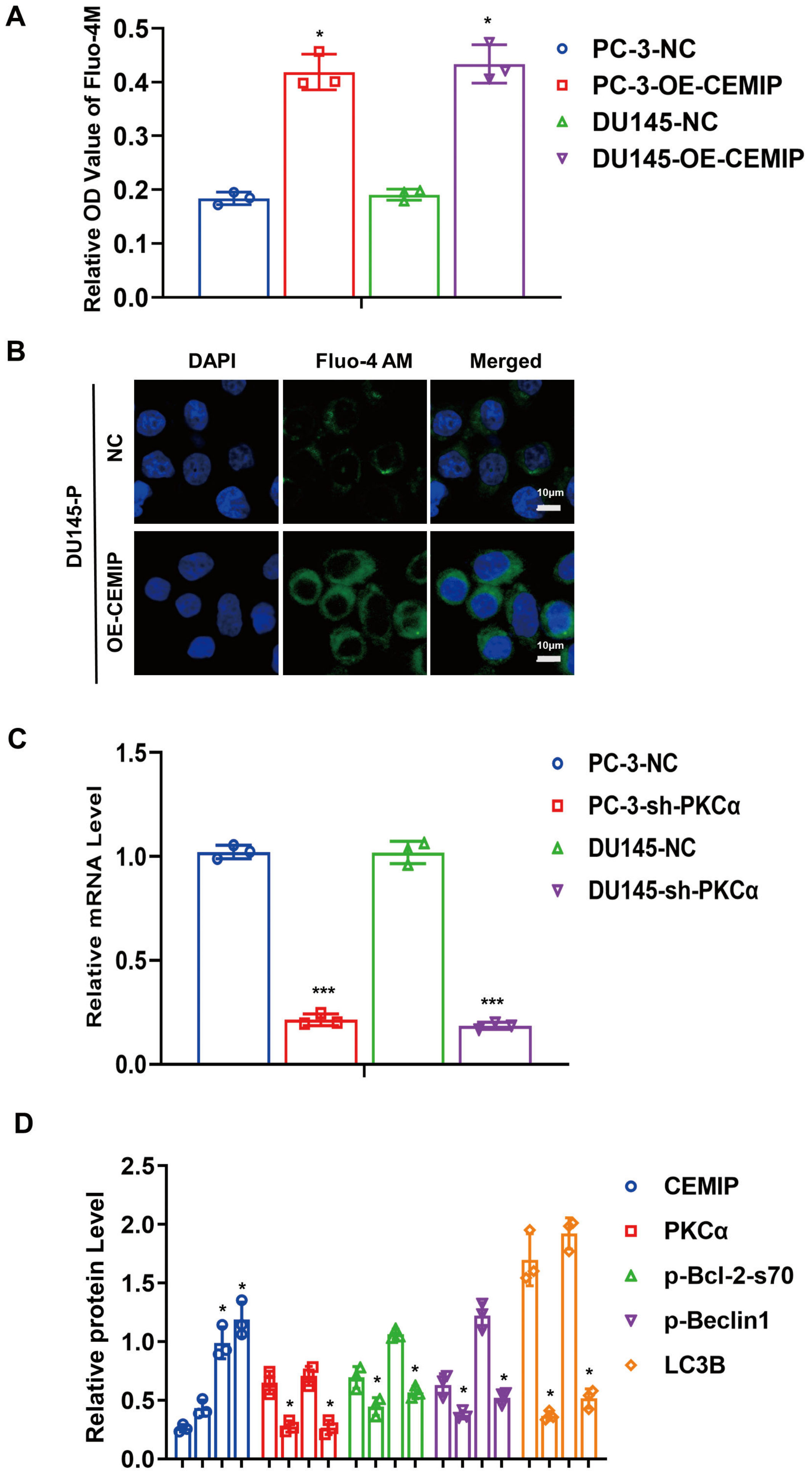
A
Relative OD Value of Fluo-4M
0.0
0.1
0.2
0.3
0.4
0.5
*
*
PC-3-NC
PC-3-OE-CEMIP
DU145-NC
DU145-OE-CEMIP
B
DAPI
Fluo-4 AM
Merged
DU145-P
NC
OE-CEMIP
10μm
10μm
C
Relative mRNA Level
0.0
0.5
1.0
1.5
***
***
PC-3-NC
PC-3-sh-PKCα
DU145-NC
DU145-sh-PKCα
D
Relative protein Level
0.0
0.5
1.0
1.5
2.0
2.5
CEMIP
PKCα
p-Bcl-2-s70
p-Beclin1
LC3B

Fig. S8

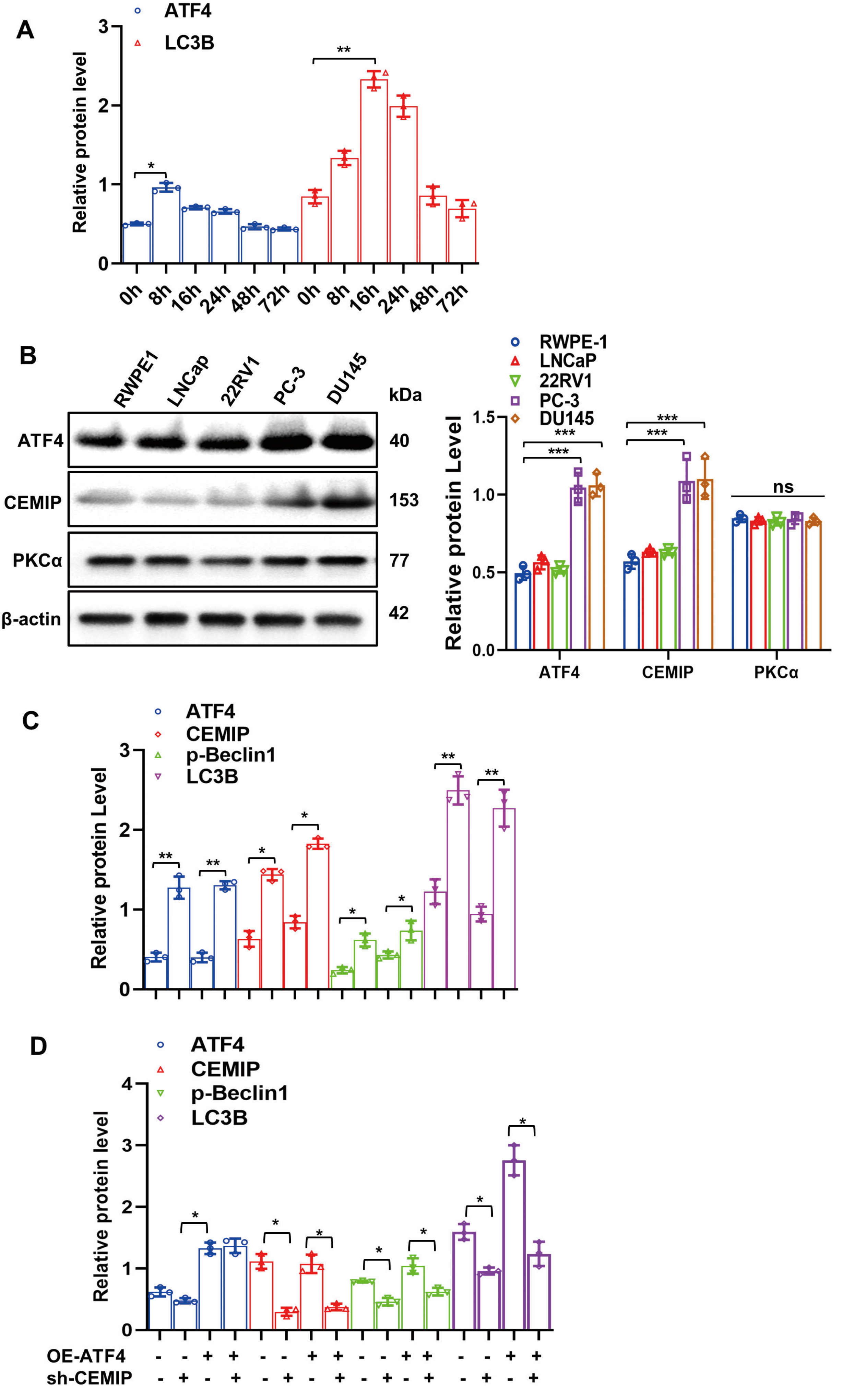
A
ATF4
LC3B
Relative protein level
0
1
2
3
*
**
0h
8h
16h
24h
48h
72h
0h
8h
16h
24h
48h
72h
B
RWPE1
LNCap
22RV1
PC-3
DU145
kDa
ATF4
40
CEMIP
153
PKCα
77
β-actin
42
RWPE-1
LNCaP
22RV1
PC-3
DU145
Relative protein Level
0.0
0.5
1.0
1.5
***
ns
ATF4
CEMIP
PKCα
C
ATF4
CEMIP
p-Beclin1
LC3B
Relative protein Level
0
1
2
3
**
*
D
ATF4
CEMIP
p-Beclin1
LC3B
Relative protein level
0
1
2
3
4
*
OE-ATF4
- - + + - - + + - - + + - - + +
sh-CEMIP
- + - + - + - + - + - + - + - +

Figure S1: Anoikis-resistant PCa cells enhance the capabilities of survival and migration.

(A) Flow cytometry assay demonstrated the rate of detachment-induced apoptosis in PCa-P and PCa-AR cells.

(B) Wound scratch assay indicated the abilities of migration in parental and anoikis resistant PC-3 and DU145 cells. (Original magnification, ×200).

(C) The migration and invasion capabilities of the parental and anoikis-resistant PCa cells were evaluated by transwell migration and matrigel invasion assays. (Original magnification, ×200).

Data are presented as the means ± SD of three independent experiments; *$p < 0.05$, **$p < 0.01$.

Figure S2: Anoikis-resistant PCa cells promote the activity of autophagy.

(A) Immunofluorescence staining demonstrated the level of autophagic flux in parental and anoikis-resistant DU145 cells. Autophagy flux were detected in 2D culture, and a total of more than 20 cells under each condition were counted for quantification of autophagy (Scale bar, 50 μm).

(B) RT-PCR assay analysis of the mRNA levels of ATF4, CEMIP, Beclin1 and autophagy related ATGs. *$p < 0.05$, **$p < 0.01$.

Data are presented as the means ± SD of three independent experiments; *$p < 0.05$, **$p < 0.01$.

Figure S3: Downregulation of CEMIP in anoikis-resistant PCa cells impairs the activity of autophagy.

(A) The protein expression levels of ATF4, CEMIP, Beclin1, ATG5, ATG7, ATG9 and ATG12 in PC-3 cells with CEMIP knockdown were detected by western blot analysis.

(B) The autophagic flux level of CEMIP-silenced DU145-AR cells was presented by immunofluorescence staining. Autophagy flux were detected in 2D culture, and a total of more than 20 cells under each condition were counted for quantification of autophagy (Scale bar, 50 μm).

(C) Western blot analysis of the reversion efficiency in autophagy of CEMIP in PC-3-AR cells on the autophagy related proteins (Beclin1, p-Beclin1, LC3BII/I) after the treatment of Rapamycin or 3-MA.

(D) The protein expression levels of ATF4, CEMIP, PKCα and Beclin1 were detected by immunohistochemistry in the lung metastases of CEMIP knockdown nude mice. Data are presented as the means ± SD of three independent experiments; $*p < 0.05$, $**p < 0.01$.

Figure S4: Downregulation of CEMIP attenuates cell survival and migration in anoikis-resistant PCa cells.

(A) Cell viability of CEMIP-downregulated in PCa-AR cells was detected by CCK-8 assay.

(B) Flow cytometry assay certified the detachment-induced apoptosis rate of downregulation of CEMIP in PCa-AR cells.

(C) Wound scratch assay showed the migration capabilities of CEMIP-downregulated in anoikis-resistant PC-3 and DU145 cells (Original magnification, ×200).

(D) The migration and invasion abilities of CEMIP downregulated in anoikis-resistant PC-3 and DU145 cells were evaluated by transwell migration and matrigel invasion assays. (Original magnification, ×200).

Data are presented as the means ± SD of three independent experiments; *$p < 0.05$, **$p < 0.01$.

Figure S5: Overexpression of CEMIP enhances migration and survival in parental PCa cells.

(A) The cell survival ability in CEMIP-overexpressing PC-3-P and DU145-P cells was demonstrated by cell viability assay.

(B) The detachment-induced apoptosis rate in CEMIP-overexpressing PCa-P cells was detected by flow cytometry assay.

(C) Wound scratch assay certified the migration capabilities of CEMIP-overexpressing PC-3 and DU145 cells (Original magnification, ×200).

(D) Transwell migration and matrigel invasion assays certified the abilities of migration and invasion in CEMIP overexpressing PC-3 and DU145 cells. (Original magnification, ×200).

(E) H&E staining demonstrated that CEMIP-overexpressed in PCa cells led to increased lung metastatic colonies. (n = 5 per group, Original magnification, ×10, ×100, respectively).

(F) Bioluminescence in vivo imaging showed the number of pulmonary metastasis focuses in CEMIP-overexpressed group and negative control group (n = 5 per group).

(G) The protein expression levels of ATF4, CEMIP, PKCα and Beclin1 were detected by immunohistochemistry in the lung metastases of CEMIP overexpressed nude mice.

Data are presented as the means ± SD of three independent experiments; *$p < 0.05$.

Figure S6: Overexpression of CEMIP enhances protective autophagy in PCa cells.

(A) qRT-PCR analysis of the mRNA level of CEMIP, Beclin-1, and autophagy related ATGs in CEMIP-overexpressing PC-3 and DU145 cells.

(B) The protein expression levels of CEMIP, Beclin1, p-Beclin1, LC3BII/LC3BI, ATG5, ATG7, ATG9, ATG12 and in PC-3-P and DU145-P cells overexpressing CEMIP were detected by western blot analysis.

(C) Immunofluorescence staining certified the level of autophagic flux in CEMIP overexpressing PC-3 and DU145 cells. Autophagy flux were detected in 2D culture, and a total of more than 20 cells under each condition were counted for quantification of autophagy (Scale bar, 50 μm).

(D) The number of double-membrane autophagosomes in CEMIP-overexpressing PC-3 cells were detected by transmission electron microscopy. (Original magnification, ×1000, ×1600, respectively).

(E) Rescue cell viability assay proved the reversion efficiency of cell survival rate by treating with rapamycin or 3-MA after CEMIP overexpressed in PC-3-P and DU145-P cells.

(F) Western blot analysis of the proteins level of CEMIP, p-Beclin1 and LC3BII/LC3BI ratio in CEMIP-overexpressing DU145-P cells after the treatment of Rapa or 3-MA.

Data are presented as the means ± SD of three independent experiments; $*p < 0.05$, $**p < 0.01$, $***p < 0.001$.

Figure S7: Overexpression of CEMIP enhances PKCα membrane translocation by aggregating of calcium ions in the cytoplasm.

(A) Immunofluorescence staining demonstrated the levels of cytoplasmic calcium ions in CEMIP-overexpressing and parental DU145 cells. Nuclei were stained blue by DAPI, cytoplasmic calcium ions were stained by Fluo4AM (Scale bar, 10 μm).

(B) qRT-PCR assay verified the effective downregulation of CEMIP after transfection for 48h in PC-3 and DU145 cells.

(C) The levels of cytoplasmic calcium ions in CEMIP overexpressing PC-3 and DU145 cells were shown by Enzyme linked immunosorbent assay after the treatment of the calcium ion probe (Fluo-4 AM) was incubated at 37°C for half an hour.

(D) The protein expression levels of CEMIP, PKCα, Beclin1, p-Beclin1, p-Bcl-2-S70 and LC3BII/LC3BI in PC-3 cells co-transformed with overexpressed CEMIP and knockdown PKCα were quantified by histogram.

Data are presented as the means ± SD of three independent experiments; $*p < 0.05$, $**p < 0.01$.

Figure S8: Upregulation of ATF4 promotes CEMIP transcription and autophagy activation

(A) Quantification of ATF4 and LC3BII/LC3BIprotein levels at different suspension time points in PC-3 cells.

(B) The protein expression levels of ATF4, CEMP and PKCα in prostate epithelial cells (RWPE-1) and four common prostate cancer cell lines (LNCaP, 22RV1, PC-3, DU145) were detected by Western blot assay.

(C) Quantification of CEMIP, p-Beclin1 and LC3BII/LC3BI protein levels in PC-3-P cells after stable overexpression of ATF4.

(D) Protein levels of ATF4, CEMIP, p-Beclin1 and LC3B were quantified after down-regulation of CEMIP in PC-3-P cells with stable overexpression of ATF4.

Data are presented as the means ± SD of three independent experiments; $^{*}p < 0.05$, $^{**}p < 0.01$, $^{***}p < 0.001$.

Supplementary Table 1: qRT-PCR primer sequences in this study

Gene names	Primer sequences
GAPDH-F	GGTCGGAGTCAACGGATTTG
GAPDH-R	GGAAGATGGTGATGGGATTTC
ATF4-F	CTCCGGGACAGATTGGATGTT
ATF4-R	GGCTGCTTATTAGTCTCCTGGAC
ATG5-F	AAAGATGTGCTTCGAGATGTGT
ATG5-R	CACTTTGTCAGTTACCAACGTCA
ATG7-F	ATTGCTGCATCAAGAAACCC
ATG7-R	GATGGAGAGCTCCTCAGCA
ATG12-F	TAGAGCGAACACGAACCATCC
ATG12-R	CACTGCCAAAACACTCATAGAGA
Beclin1-F	AGGATGATGTCCACAGAAAGTGC
Beclin1-R	AGTGACCTTCAGTCTTCGGCTG
ATG9L1-F	TGTTTCTCAATGAATGGAGCCTC
ATG9L1-R	AAGTTAGCGATGCCAATCCAC
ATG14L-F	GCAAATCTTCGACGATCCCAT
ATG14L-R	CACACCCGTCTTTACTTCCTC
ATG16L1-F	GGCAGTAGCTGGTACCCTCACTTC
ATG16L1-R	CTCTGTCTCTTCCTTCCCAGTCC
PKCα-F	TGGACTTATCCATCAAGGGATGA
PKCα-R	AGTGTGATCCATTCCGCAGAG
BCL2-F	GGTGGGGTCATGTGTGTGG
BCL2-R	CGGTTCAGGTACTCAGTCATCC
CEMIP-F	GGCCGGTGATGTAGACGAAA
CEMIP-R	CCATTGGAGCCATGGACTGT

Supplementary Table 2: Primary and secondary antibodies

Antibody	Source	Identifier	Host
CEMIP antibody	Abcam	ab62322	Rabbit
ATF4 antibody	Proteintech	10835-1-AP	Rabbit
PKCα antibody	Proteintech	21991-1-AP	Rabbit
Bcl-2 antibody	CST	15071S	Mouse
p-Bcl-2 antibody	CST	2827S	Rabbit
Beclin1	CST	3738S	Rabbit
p-Beclin1	CST	14717S	Rabbit
LC3B	Abcam	ab192890	Rabbit
β-actin	Proteintech	66009-1-Ig	Mouse
488 - Anti-Mouse IgG(H+L)	Proteintech	SA00013-1	Mouse
594 –Anti-Rabbit IgG(H+L)	Proteintech	SA00013-4	Rabbit
ATG5	Proteintech	10181-2-AP	Rabbit
ATG7	Proteintech	10088-2-AP	Rabbit
ATG9	Proteintech	26276-1-AP	Rabbit
ATG12	Proteintech	11122-1-AP	Rabbit

Supplementary Table 3: Drugs and reagents

Drug / Reagent	Source	Identifier/ formulation
FITC-Annexin V apoptosis	BD Biosciences kit	556547
PE-Annexin V apoptosis detection	BD Biosciences kit	559763
CCK-8	Vazyme Biotech	A311-01
rapamycin	Selleck	S1039
3-Methyladenine	Selleck	S2767
Chromatin immunoprecipitation	Beyotime (ChIP) kit	P2078
7-AAD	BD Biosciences kit	559925

Supplementary Table 4: Sequence of Bcl-2 mutations in the study

Human Bcl-2 point mutations that mimic phosphorylation sequences
ATGGCGCACGCTGGGAGAACAGGGTACGATAACCGGGAGATAGTGATGA
AGTACATCCATTATAAGCTGTCGCAGAGGGGCTACGAGTGGGATGCGGG
AGATGTGGGCGCCGCGCCCCCGGGGGCCGCCCCCGCACCGGGCATCTTC
TCCTCCCAGCCCGGGCACACGCCCCATCCAGCCGCATCCCGGGACCCGG
TCGCCAGGACC**GAT**CCGCTGCAGACCCCGGCTGCCCCCGGCGCCGCCGC
GGGGCCTGCGCTCAGCCCGGTGCCACCTGTGGTCCACCTGACCCTCCGC
CAGGCCGGCGACGACTTCTCCCGCCGCTACCGCCGCGACTTCGCCGAGA
TGTCCAGCCAGCTGCACCTGACGCCCTTCACCGCGCGGGGACGCTTTGC
CACGGTGGTGGAGGAGCTCTTCAGGGACGGGGTGAACTGGGGGAGGAT
TGTGGCCTTCTTTGAGTTCGGTGGGGTCATGTGTGTGGAGAGCGTCAAC
CGGGAGATGTCGCCCCTGGTGGACAACATCGCCCTGTGGATGACTGAGT
ACCTGAACCGGCACCTGCACACCTGGATCCAGGATAACGGAGGCTGGGA
TGCCTTTGTGGAACTGTACGGCCCCAGCATGCGGCCTCTGTTTGATTTCT
CCTGGCTGTCTCTGAAGACTCTGCTCAGTTTGGCCCTGGTGGGAGCTTGC
ATCACCCTGGGTGCCTATCTGGGCCACAAGTGA

Supplementary Table 5: Sequences of knockdown in the study

**Gene names**	**Sequences**
CEMIP shRNA1	CCAGGAATGTTGAATGTCTTTTTCAAGAGAAAAGACA TTCAACATTCCTGGTTTTTT
CEMIP shRNA2	CGAATGAAGATCATCAAGAATTTCAAGAGAATTCTTG ATGATCTTCATTCGTTTTTT
CEMIP shRNA3	GATCCTTACTATGGTCTGATTCAAGAGATCAGACCATA GTAAGGATCTTTTTT
PKCα shRNA1	GCGTCCTGTTGTATGAAATTTCAAGAGAATTTCATACA ACAGGACGCTTTTTT
PKCα shRNA2	AAGCTCCATGTCACAGTACGATTCAAGAGATCGTACT GTGACATGGAGCTTTTTTTT
PKCα shRNA3	CCATCCGCTCCACACTAAATTCAAGAGATTTAGTGTGG AGCGGATGGTTTTTT

Supplementary Table 6: Predicted ATF4 binding sites on CEMIP promoter

NC_000015.10: 80777273-80779525 Homo sapiens chromosome 15, RCh38.p12
ATGGATTGCGAAAGTGTGGGCAGTAAATCCACCAGTG***TCATGTTGCTATA***T<br>TTATCCATAAAGAATTACGCGGCACGCCCCGTTCATCACTGAGTGCTGAAT<br>GGCAGTGCTGAATGGAGAACTGGCTGGAGAATCTGGTTTTAGACATTCGG<br>AGCCTGTTTG***TAATTCATCAGCA***CTGCTCTTTTCCCCTGGGCATTGGTCTGT<br>GATAAAGAAAACTGGATACCAGAGGAATGTGCTGGGCATTTCAGTGCTTT<br>GAGAAAACATAGCCAAGCACTTCTCAGGGCCCATCCCTAGACAGGACCCT<br>GCCTTTACCACTTCATCTCCCAGATGGCTGTGGAGCCAGCCCCTCACCAGA<br>GAGCTACCTGGCTCCACTCAGGCCTCAAATCTACTGTCTGTTGATGGAAAA<br>TCCTGCATGTCGTGCCAAGCCA***GGCTGATTCAAGG***TCGACCTTTCTCCATT<br>TGGGCCAAATGCCCAGCACCCCCAAACCCCTAACACACAACTCTTTGTGG<br>GTCTGGTTTCTGGGAGCTCCTTAAGGAAGAAAAACATAAACAGGAATCAT<br>GGTGAGGACTGGGGAGAGGGGCCCTGGGTCGGGGGGGCTCCATTTTATTC<br>GGCTGGAAAGAAAAGGTTGGGGAGGAGGGCTTGCCATTGGATCCAGTTC<br>ATTACTTCCCAATAAATCAAAGAAGGGCTGAGAATCCTGACCACCACCCCC<br>AGTAAGGATCAAGTTCCGCTTTCTGGAAGAGGCCTAGAGGTGGATTGCAG<br>GACTTCCCAGTTTAACCAGGTGACAAAGGCTTCTGCTTCAGCCCAGATGT<br>GATCACAGACTTCCACAGCTTCTGCCGACCACAGCCTGGCCTTAAAGTCC<br>AGACCTAGAGTCCCGGAGCTAGTGTGTACTACACAGAATGGAGACCTTGC<br>CTGAGGAGCTGGTTCTCAGAGAACACTGGCAGGAAGGAAGAAAGAAATG<br>TATATTCTAGGAGGCCTCTGCTAACCAGGAAAGGGGGCTGAGTCACACTCT<br>GAAAGGTGGAAGAGGCAGAGAGCAGGTATCAACGCCCTCATTTTACAGAT<br>TAAAACAAAACAAGAAAAACAAACACTCCTCACCTCCCTGCAAAAAAAC<br>CACCCTGAGGCTCAGACAGGGGAAGTAGCTGGCCCTGGTCACACAGTAG<br>GTTAGCGGCAGAGTTGAGAGGAGACTCCATATGCCCTAGGGATGTGTTGT<br>GATGAACTTTTCCTACTGGTACTGTTTCCTCCCGCGAGGGAATGTCTAGAC<br>CAGCCGCACCTTCTTGCTTTGACCCTTCAGAACTTTGGCCTGTCCAGTTAA<br>AGAGGCACAGAGCCCTCCTACCCACAGGGAATGTTTCTAACTTACCAAGC

ACCTATTATGTGCTTAGAACTTGGACTTTCCTGATCTCATTTAAGCCTCACC
ATTCTAGTGAGGAAGCATCACAGAACCCATTTTCC***TGATGAGGAAACT***GAG
ACTGGGAGGGGTTAAGATCCTGTACACAGTTGTAACTGGCATGTCTGAATC
CAGTTTCAAACCAAGGTGCCTTTCAGTGAACACCCAAGCATCTTCCTCTC
GGATTAACTGCTAGGCCAATACCAAAATCTCTTTCTCGGGCTACTCTTTTGC
TGCTGAGTTAAAAAGAGATCCTGAGACTTAGCCTGGGACCCCGACTCGCG
GCGATTCTGTGGCAGGCGCAGACCTTTTCTCCTGTCCTCCACTGCCTCTTT
GCCTCGGAGGGCGCCCCGGGGCGCGAGCGGGGCACGGGCGACCCTGGCT
CCTTGGAGGCCCGGGGACTTGCTCTCCGCGGTCCGCCGCGGCCGCTCCAC
CGAGTCCTGGCCTCCAGCGCCCTCACCTGGCTGCTGCGGGCGGGACTCTG
CGCACCCGGGCTCCGCGGGCTCAGGTCTGCGTCTCCCAGAGCCGCCGCCA
GAGCCCGCCCCGGTCCGGCCCGGGCACAGGGCCCCCGCCCCACTTGGGC
AAGGCGGGGGCGCGTGGAGGGAAGTTTCATCGGGGGGCGAGGGAGGGA
GCTTCGGTGCCGCGCGGGCGTCAGGGGCTCCTCGGGGGAGGAGTCAGTA
AAAGTGGCTATAAAAGCGGCCTCTCGGCTGAGGGCCGGGGAGCTAGCGCT
CAAGCAGAGCCCAGCGCGGTGCTATCGGACAGAGCCTGGCGAGCGCAAG
CGGCGCGGGGAGCCAGCGGGGCTGAGCGCGGCCAGGGTCTGAACCCAGA
TTTCCCAGACTAGCTACCACTCCGCTTGCCCACGCCCCGGGAGCTC

the 3 site sequence: GGCTGATTCAAGG

the 3 site mutant: GGCCCGTCCGAAG
